# Combination of IL-17A/F and TNF-α uniquely alters the bronchial epithelial cell proteome to enhance proteins that augment neutrophil migration

**DOI:** 10.1186/s12950-022-00323-w

**Published:** 2022-12-14

**Authors:** Anthony Altieri, Hadeesha Piyadasa, Mahadevappa Hemshekhar, Natasha Osawa, Breann Recksiedler, Victor Spicer, Pieter S Hiemstra, Andrew J Halayko, Neeloffer Mookherjee

**Affiliations:** 1 Manitoba Centre for Proteomics and Systems Biology, Department of Internal Medicine, University of Manitoba, Winnipeg, MB Canada; 2 Department of Immunology, University of Manitoba, Winnipeg, MB Canada; 3 Department of Pathology, School of Medicine, Stanford University, Palo Alto, CA USA; 4 Department of Pulmonology, Leiden University Medical Center, Leiden, The Netherlands; 5 Department of Physiology and Pathophysiology, University of Manitoba, Winnipeg, MB Canada; 6 Biology of Breathing Group, The Children’s Hospital Research Institute of Manitoba, Winnipeg, MB Canada

**Keywords:** IL-17A/F, TNF-α, Inflammation, Lung, Host defence peptides, Neutrophils

## Abstract

**Background:**

The heterodimer interleukin (IL)-17A/F is elevated in the lungs in chronic respiratory disease such as severe asthma, along with the pro-inflammatory cytokine tumor necrosis factor-α (TNF-α). Although IL-17A/F and TNF-α are known to functionally cooperate to exacerbate airway inflammation, proteins altered by their interaction in the lungs are not fully elucidated.

**Results:**

We used Slow Off-rate Modified Aptamer-based proteomic array to identify proteins that are uniquely and/or synergistically enhanced by concurrent stimulation with IL-17A/F and TNF-α in human bronchial epithelial cells (HBEC). The abundance of 38 proteins was significantly enhanced by the combination of IL-17A/F and TNF-α, compared to either cytokine alone. Four out of seven proteins that were increased > 2-fold were those that promote neutrophil migration; host defence peptides (HDP; Lipocalin-2 (LCN-2) and Elafin) and chemokines (IL-8, GROα). We independently confirmed the synergistic increase of these four proteins by western blots and ELISA. We also functionally confirmed that factors secreted by HBEC stimulated with the combination of IL-17A/F and TNF-α uniquely enhances neutrophil migration. We further showed that PI3K and PKC pathways selectively control IL-17A/F + TNF-α-mediated synergistic production of HDPs LCN-2 and Elafin, but not chemokines IL-8 and GROα. Using a murine model of airway inflammation, we demonstrated enhancement of IL-17A/F, TNF-α, LCN-2 and neutrophil chemokine KC in the lungs, thus corroborating our findings *in-vivo.*

**Conclusion:**

This study identifies proteins and signaling mediated by concurrent IL-17A/F and TNF-α exposure in the lungs, relevant to respiratory diseases characterized by chronic inflammation, especially neutrophilic airway inflammation such as severe asthma.

**Supplementary Information:**

The online version contains supplementary material available at 10.1186/s12950-022-00323-w.

## Background

Interleukin (IL)-17 is a critical mediator of airway inflammation, associated with the development and increased severity in chronic respiratory disease [1–3]. IL-17 levels are significantly higher in patients with severe asthma, in the disease phenotype that cannot be effectively controlled with available treatments [3–10]. A challenge in the development of new treatments is the lack of a comprehensive understanding of the range of molecular changes orchestrated by the interplay of IL-17 with other cytokines that are enhanced in the lungs during chronic inflammatory respiratory disease.

The IL-17 family of cytokines includes six different members. The highly homologous IL-17A and IL-17F, and its heterodimer IL-17A/F, are predominantly associated with airway inflammation in humans [11, 12]. IL-17A, IL-17F and IL-17A/F are produced by multiple cell types found at mucosal surfaces of the lung, including CD4^+^ T-helper (Th)17 cells, CD8+ (Tc)17 effector cells, γδ-T cells, natural killer T cells and type 3 innate lymphoid cells-3 [12–14]. IL-17A, IL-17F, and IL-17A/F have been demonstrated to induce qualitatively similar gene activation however these are quantitatively different [15]. These cytokines bind to the dimeric IL-17RA and IL-17RC receptor complex to mediate downstream inflammatory responses [16, 17]. IL-17RA is ubiquitously expressed, but IL-17RC is primarily restricted to non-hematopoietic cells [18, 19]. During airway inflammation the activation of the IL-17RA/RC receptor complex in structural cells, such as airway epithelial cells, results in the production of known IL-17 downstream targets which includes pro-inflammatory cytokines, chemokines, airway remodeling factors, and host defence peptides (HDP) with antimicrobial functions [14, 18, 20, 21]. Although many of these downstream targets have been previously defined, these were primarily characterized for IL-17A, but not for the heterodimer IL-17A/F.

The biological activity of IL-17A, IL-17F, and the heterodimer IL-17A/F is increased in asthma [3, 5, 8, 9, 22, 23]. A previous study demonstrated that mucosa airway biopsies of patients with severe asthma have increased expression of both IL-17A and IL-17F [5]. While IL-17F-producing Th17 cells are increased in the lung submucosa of both mild-moderate and severe asthmatics, IL-17A-producing Th17 cells are only increased in mild-moderate asthmatic subjects [23]. These studies suggest that the heterodimer IL-17A/F is more likely to be enhanced in severe asthma, compared to IL-17A alone. In severe asthma, although the heterodimer IL-17A/F is known to interplay with other cytokines enhanced in the lungs such as TNF-α [24, 25], the downstream targets, signaling intermediates and functional outcomes of this interaction remain largely unknown. Thus, the aim of this study was to define global protein changes and signaling intermediates mediated by the heterodimer IL-17A/F, and how these responses change in the presence of TNF-α, in bronchial epithelial cells.

We have previously demonstrated that IL-17A/F and TNF-α alone disparately alter specific antimicrobial proteins and peptides, and various chemokines in human bronchial epithelial cells [21]. Therefore, in this study we comprehensively characterized the human bronchial epithelial cellular proteome altered by IL-17A/F, in the presence and absence of TNF-α. We further independently confirmed the abundance of selected proteins, performed functional validation, and examined mechanistic signaling pathways, involved in the combinatorial effect of IL-17A/F and TNF-α in human bronchial epithelial cells. As in our previous study we had established that IFN-γ mediated changes in the abundance of antimicrobial peptides and chemokines are distinctly different from that elicited by either IL-17A/F or TNF-α [21], we used IFN-γ as a paired control along with IL-17A/F and TNF-α, for stimulation of human bronchial epithelial cells. We also confirmed the induction of selected proteins uniquely induced by the combination of IL-17A/F and TNF-α in a murine model of airway inflammation. Overall, the findings in this study provide a comprehensive assessment of downstream protein targets and signaling intermediates mediated by the combinatorial effect of IL-17A/F and TNF-α, and confirms its relevance in the augmentation of neutrophilic airway inflammation.

## Results

### IL-17A/F and TNF-α combination uniquely alters the bronchial epithelial cell proteome

**Fig. 1:**
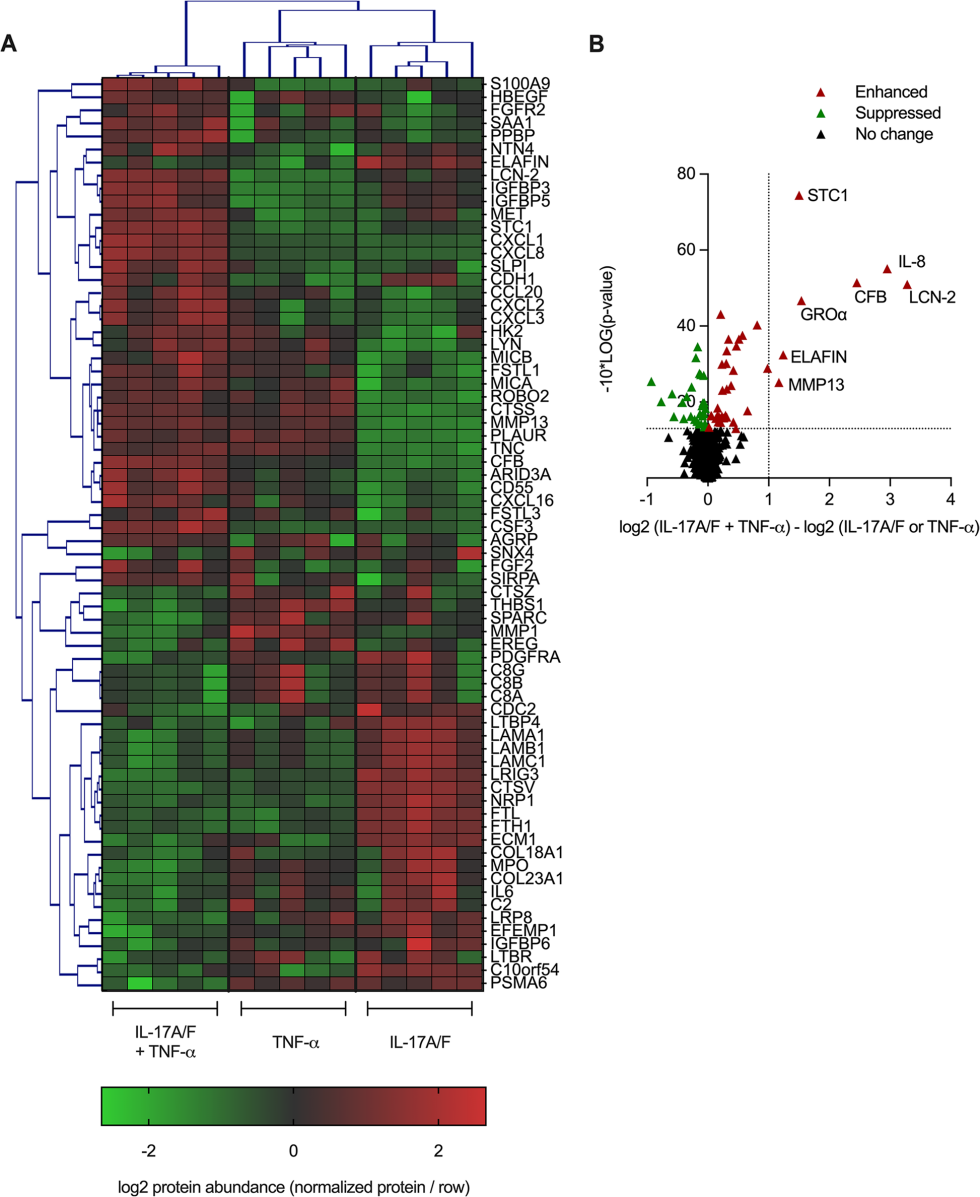
Characterization of the human bronchial epithelial cell proteome. HBEC-3KT were stimulated with IL-17A/F (50 ng/mL) in the presence and absence of TNF-α (20 ng/mL) for 24 h. Cell lysates (14 µg total protein per sample) obtained from five independent experiments were probed using the Slow off-rate Modified Aptamer proteomic array. Increases in log2 protein abundance in response to IL-17A/F, TNF-α, or IL-17A/F + TNF-α was calculated after subtraction of background values in paired unstimulated cells. Pairwise differential analysis was conducted on normalized log2 protein expression values, and Welch’s t-test with a cutoff of *p* < 0.05 was used to select proteins that were significantly enhanced in response to the combination of IL-17 A/F + TNF-α, compared to either cytokine alone. Log2 protein abundance values were normalized per row in the heat map to yield a consistent dynamic range for visualization. (**A**) Heat map generated using Multi-Experiment Viewer Version 10.2 to visualize protein expression profile, where each column represents an independent experiment (*n* = 5 per condition). (**B**) Volcano plot demonstrating differentially abundant proteins in response to the combination of IL-17A/F and TNF-α, compared to either cytokine alone

Human bronchial epithelial cells (HBEC)-3KT (ATCC CRL-4051) were stimulated with IL-17A/F (50 ng/mL), in the presence and absence TNF-α (20 ng/mL) or IFN-γ (30 ng/mL), for 6, 12, 24 and 48 h. Cytokine concentrations were selected based on previous studies [3, 9, 21, 26]. Chemokines GROα, IL-8 and MCP-1 production was examined in the tissue culture (TC) supernatants by ELISA (Supplementary Fig. 1). Kinetics of chemokine response showed that all three chemokines were significantly enhanced after 24 h stimulation (Supplementary Fig. 1), and thus 24 h time point was selected for the proteomics study. Cell lysates (14 µg total protein per sample) were obtained from five independent experiments of HBEC-3KT cells stimulated with IL-17A/F (50 ng/mL) in the presence and absence TNF-α (20 ng/mL), or IFN-γ (30 ng/mL) as a paired control, for 24 h. Each lysate was independently probed using the Slow Off-rate Modified Aptamer proteomic array (*n* = 5 for each group). Pairwise differential analysis conducted on normalized log2 protein abundance values showed that IL-17A/F + TNF-α cytomix significantly altered (*p* < 0.05) the abundance of 70 proteins, compared to either cytokine alone (Supplementary Table I). In contrast, IL-17A/F did not significantly alter IFN-γ-mediated protein production in HBEC-3KT (data not shown). Hierarchical clustering of the 70 proteins identified to be uniquely altered with the combination of IL-17A/F + TNF-α showed a distinct protein profile compared to either cytokine alone (Fig. 1 A). Of these 70 proteins, IL-17A/F + TNF-α cytomix increased the abundance of 38 proteins and decreased the abundance of 32 proteins, compared to either cytokine alone (Supplementary Table I). The 38 proteins that were significantly enhanced by the combination of IL-17A/F + TNF-α were primarily associated with three functional categories: HDP, neutrophil chemotactic factors, and airway remodeling factors. In addition, bioinformatics assessment of proteins that were enhanced by IL-17A/F + TNF-α compared to either cytokine alone, using an in-house analytical tool developed to compute enrichment specific to the SOMAmer®-based collection of > 1300 proteins, identified biological processes which drive neutrophil accumulation in the lungs, such as neutrophil chemokine receptor binding, positive regulation of neutrophil chemotaxis, and chemokine-mediated signaling pathways, as overrepresented biological pathways (Supplementary Fig. 2). Seven of the 38 proteins were significantly increased by ≥ 2-fold, compared to either cytokine alone (Fig. 1B). Of these seven proteins, five belonged to the above mentioned three functional categories of HDP (Lipocalin 2 (LCN-2) and Elafin), neutrophil chemokines (IL-8 and GROα), and airway remodeling factor matrix metalloproteinase 13 (MMP13). Therefore, we selected these five proteins for further independent confirmatory and mechanistic studies. Four out of the five selected proteins (LCN-2, Elafin, GROα and IL-8) are also known to enhance neutrophil migration at mucosal surface [27–30], albeit LCN-2 and Elafin have been predominantly described in the context of antimicrobial functions [28, 31, 32]. Based on these results, and our previous study demonstrating that the protein expression profile mediated by IFN-γ is distinctly different from either IL-17A/F or TNF-α [21], we used IFN-γ as a paired negative control in subsequent in vitro studies as follows.

### IL-17A/F and TNF-α combination synergistically enhances transcription of neutrophil chemokines and Lipocalin-2

**Fig. 2:**
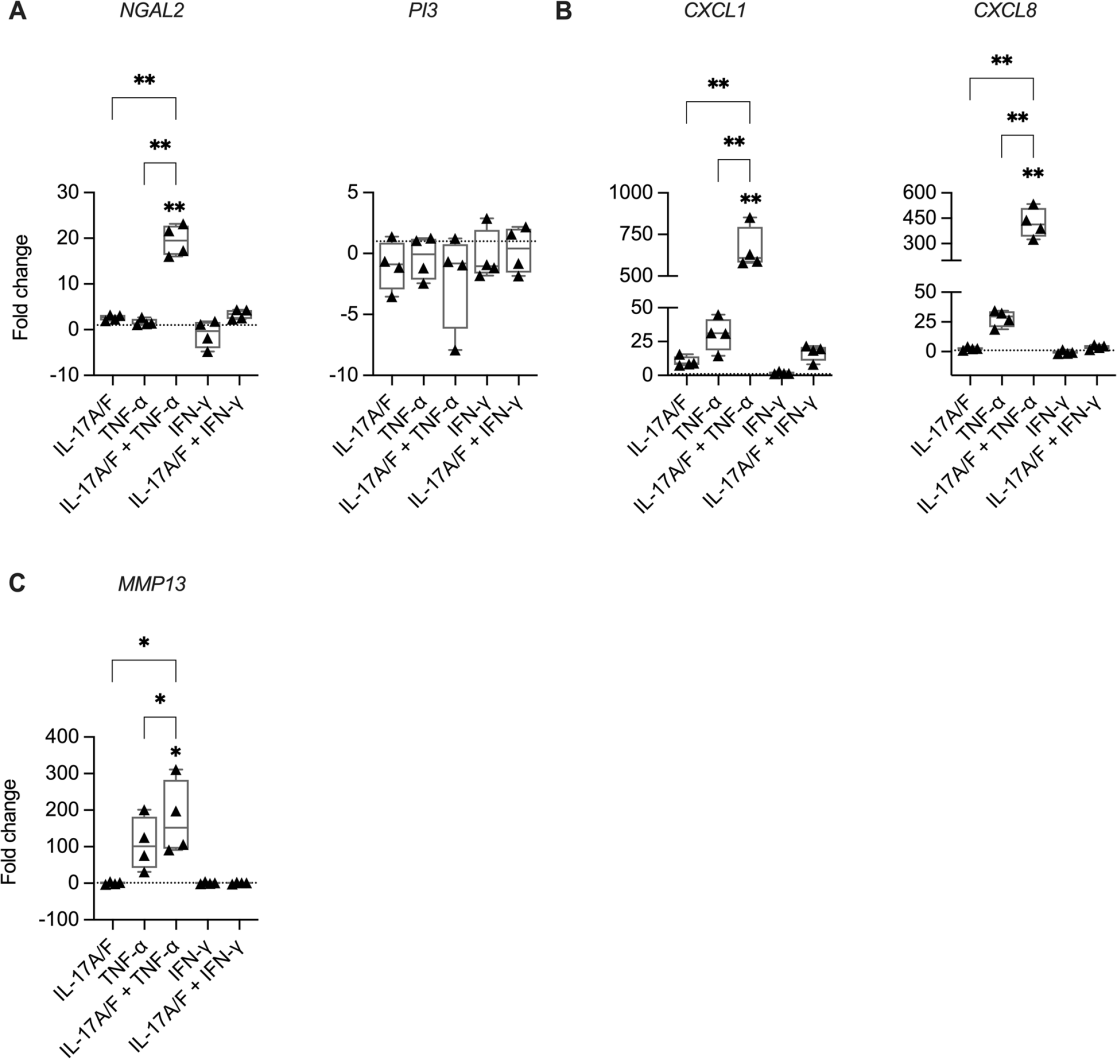
Independent validation of transcriptional responses of selected protein targets. HBEC-3KT were stimulated with either IL-17A/F (50 ng/mL), TNF-α (20 ng/mL) or IFN-γ (30 ng/mL), or cytokines combinations as indicated, for 6 h. mRNA was isolated and transcriptional responses evaluated by quantitative real-time PCR for (**A**) antimicrobial HDP LCN-2 *(NGAL2)* and Elafin *(PI3)*, (**B**) neutrophil chemokines GROα (*CXCL1*) and IL-8 (*CXCL8*), and **(C)***MMP13*. Fold changes (y-axis) for each gene was normalized to 18S RNA, and calculated compared to unstimulated cells normalized to 1, using the comparative ΔΔCt method. Results are shown as boxplots, wherein bars show median and IQR, and whiskers show minimum and maximum values. Each data point represents an independent experimental replicate (*n* = 4). Fisher’s LSD test for one-way analysis of variance (ANOVA) was used to determine statistical significance (*p < 0.05, **p < 0.01). The dashed line represents normalized baseline value of 1 for unstimulated cells

HBEC-3KT cells were stimulated with IL-17A/F (50 ng/mL), TNF-α (20 ng/mL) or IFN-γ (30 ng/mL), and cytomix as indicated, for 6 h. mRNA expression of genes encoding for the proteins selected from the proteomics data were examined by qRT-PCR. mRNA expression of *NGAL2* (encoding for LCN-2), but not *PI3* (gene for Elafin), was synergistically enhanced in a supra-additive manner by the combination of IL-17A/F and TNF-α (by > 19-fold) compared to unstimulated or either cytokine alone (Fig. 2 A). Expression of *CXCL1* and *CXCL8* (encoding for GROα and IL-8 respectively) were also enhanced in a supra-additive manner by the cytomix IL-17A/F and TNF-α (> 650-fold and > 400-fold respectively) compared to unstimulated cells or each cytokine alone (Fig. 2B). TNF-α alone significantly enhanced the expression of *MMP13* by ~ 100-fold compared to unstimulated cells, and this was further significantly enhanced by IL-17A/F (Fig. 2 C). Transcription of none of the selected proteins was enhanced in response to either IFN-γ or its combination with IL-17A/F. These results demonstrated that transcription of three out of the five selected proteins (LCN-2, GROα, and IL-8) was synergistically enhanced by the combinatorial action of IL-17A/F and TNF-α in HBEC.

### IL-17A/F and TNF-α combination synergistically enhances protein production of neutrophil chemokines, LCN-2 and Elafin

**Fig. 3:**
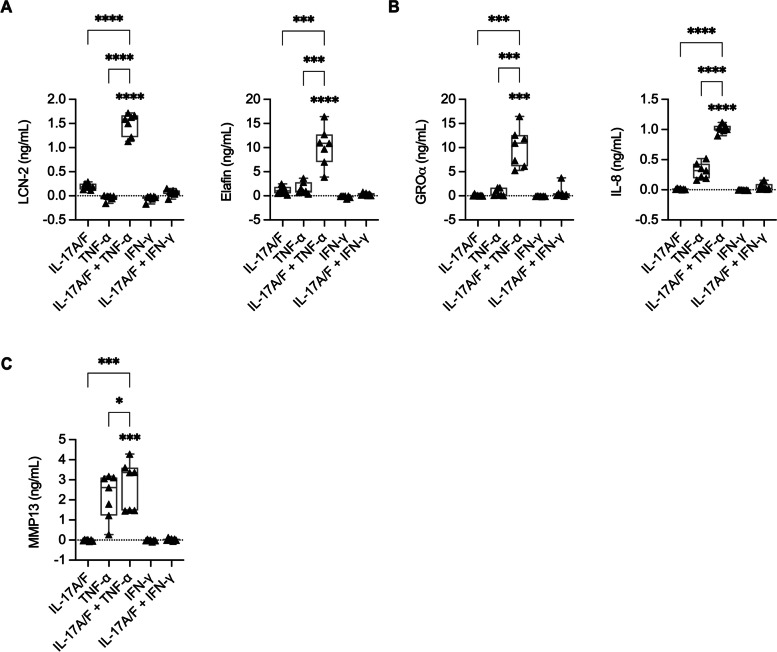
Independent validation of selected protein production in human bronchial epithelial cells. HBEC-3KT were stimulated with either IL-17A/F (50 ng/mL), TNF-α (20 ng/mL) or IFN-γ (30 ng/mL), or cytokines combinations as indicated, for 24 h. Tissue culture supernatants were examined by ELISA for the protein abundance of (**A**) antimicrobial HDP LCN-2 and Elafin, (**B**) neutrophil chemokines GROα and IL-8, and (**C**) MMP-13. Increases in protein abundance are reported after subtraction of background values in paired unstimulated cell samples per replicate. The dashed lines represent average baseline value in unstimulated cells. Results are shown as boxplots, wherein bars show median and IQR, and whiskers show minimum and maximum value. Each data point represents results an independent experimental replicate (*n* = 7). Fisher’s LSD test for one-way ANOVA was used to determine statistical significance (*p < 0.05, **p < 0.01, ***p < 0.001, ****p < 0.0001)

**Fig. 4:**
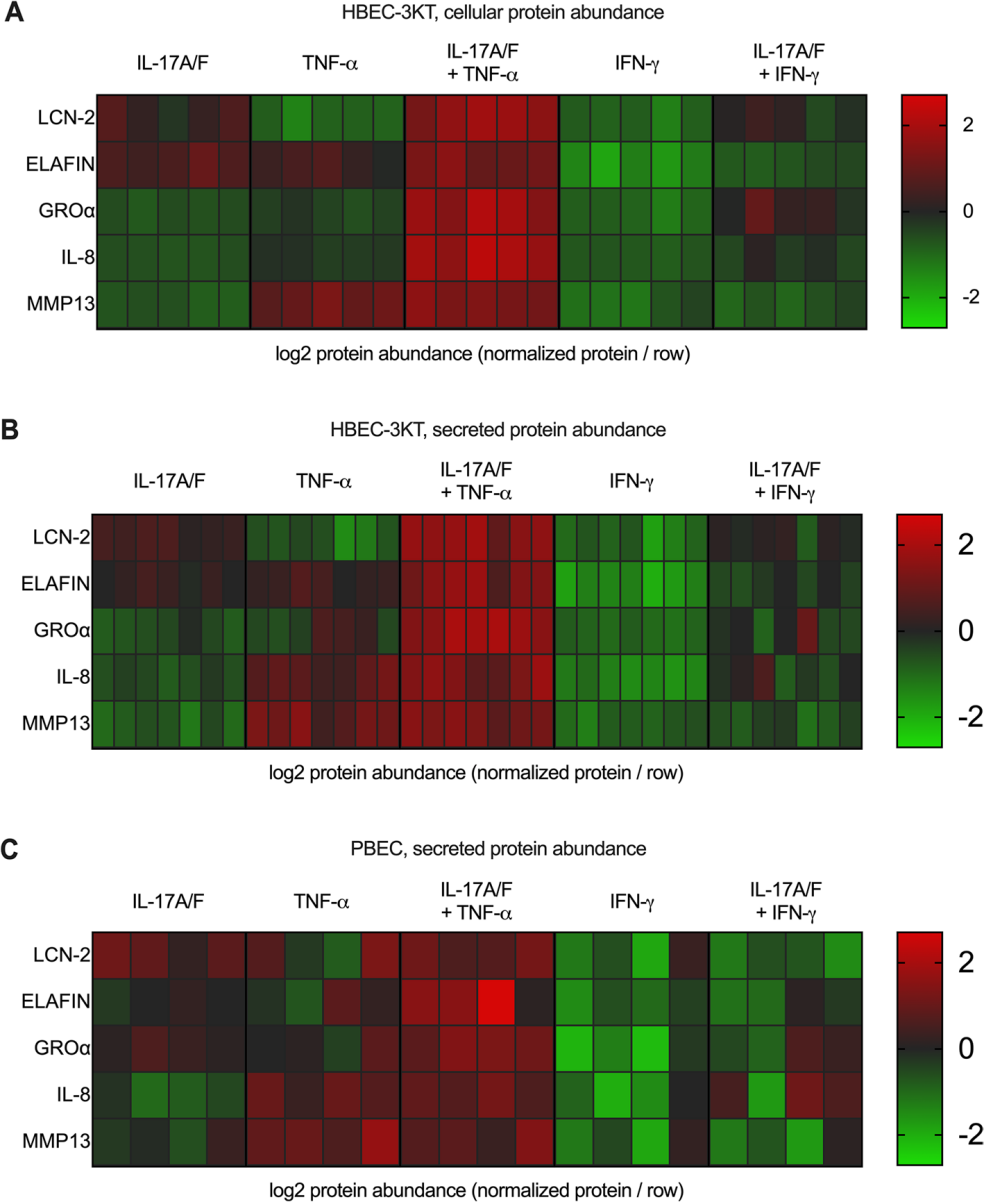
Comparative analyses of protein abundance profile in HBEC and human PBEC isolated from lungs. (**A**) HBEC-3KT (*n* = 5) were stimulated with IL-17A/F (50 ng/mL), TNF-α (20 ng/mL), IFN-γ (30 ng/mL) or cytokine combinations, as indicated for 24 h, and cell lysates were used proteomics profiling by SOMAmer®-based protein array. (**B**) HBEC-3KT (*n* = 7) and (**C**) human PBEC (*n* = 4 independent donors), were stimulated with IL-17A/F (50 ng/mL), TNF-α (20 ng/mL) or IFN-γ (30 ng/mL), or cytokine combinations, as indicated for 24 h. Tissue culture supernatants were monitored for protein abundance of LCN-2, Elafin, GROα, IL-8 and MMP13, by ELISA after 24 h. Increases in protein abundance was calculated after subtraction of background values in paired unstimulated cells for each biological replicate. Log2 protein abundance values were normalized per row in the heat map to yield a consistent dynamic range for visualization and comparative analyses

We independently examined protein production of the five proteins (LCN-2, Elafin, GROα, IL-8, and MMP13) selected from the proteomics dataset, in HBEC-3KT cells, and in human primary bronchial epithelial cells (PBEC) isolated from lung tissues of four patients undergoing lung resection. Independent western blot analyses showed that protein abundance of both HDPs, LCN-2 and Elafin, were synergistically enhanced by the cytomix IL-17A/F + TNF-α in a supra-additive manner, compared to either cytokine alone in HBEC-3KT cell lysates (Supplemental Fig. 3), thus validating the proteomics data set. As secreted proteins primarily mediate cellular communication and pathophysiological changes, we also examined the abundance of the five selected proteins by ELISA in TC supernatants obtained from HBEC-3KT cells stimulated with the cytokines or cytomix as indicated, after 24 h. Abundance of LCN-2, Elafin, GROα and IL-8 were all significantly enhanced by the combination of IL-17A/F and TNF-α in a supra-additive manner, compared to either cytokine alone, in TC supernatants obtained from HBEC-3KT cells (Fig. 3 A and 3B). In contrast, protein abundance of MMP13 was significantly increased by TNF-α alone and modestly enhanced by the cytomix IL-17A/F + TNF-α in TC supernatants (Fig. 3 C). To confirm these effects in primary cells, we further monitored the abundance of the five selected proteins in TC supernatants by ELISA obtained from human PBEC stimulated with the cytokines or cytomix as indicated, after 24 h. Log2 protein abundance values obtained from TC supernatants of PBEC and HBEC-3KT, and from the cellular proteome dataset, was normalized per row in a heat map to obtain comparable dynamic range for visualization and for comparative analyses. Protein abundance profile mediated in response to combination of IL-17A/F and TNF-α compared to either cytokine alone, in HBEC-3KT cellular proteome (Fig. 4 A) was similar to that observed in the TC supernatants from HBEC-3KT (Fig. 4B) and PBEC (Fig. 4 C), with the exception of IL-8 production which was preferentially driven by TNF-α in PBEC. Taken together, our results demonstrated that the protein production of LCN-2, Elafin, IL-8, and GROα are synergistically enhanced by the combinatorial effect of IL-17A/F and TNF-α, and that these increased protein abundance are also found in the extracellular milieu.

### LCN2 and Elafin production mediated by the combination of IL-17A/F and TNF-α involves PKC and PI3K signaling pathways

**Fig. 5:**
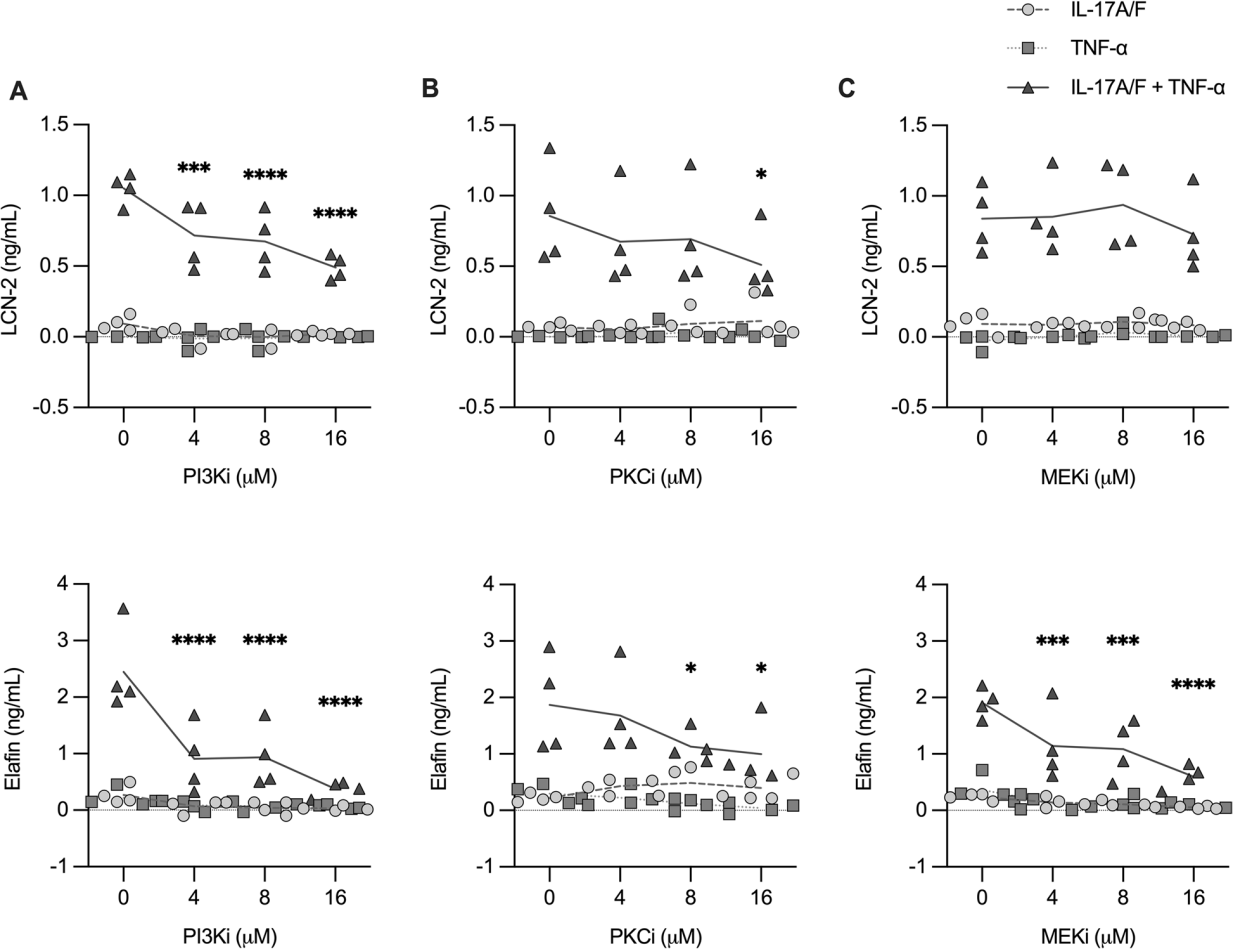
Assessment of pharmacological inhibitors on IL-17A/F and TNF-α mediated production of selected proteins. HBEC-3KT cells were pre-treated with pharmacological inhibitors (**A**) LY294002 (PI3Ki), (**B**) GO6976 (PKCi) and (**C**) PD98059 (MEKi), for 1 h prior to stimulation with IL-17A/F (50 ng/ml), TNF-α (20 ng/ml) or the combination of IL-17A/F and TNF-α. Tissue culture supernatants were collected after 24 h and examined for protein production by ELISA for LCN-2 and Elafin. Protein abundance shown is after subtraction of background abundance in paired unstimulated cells in each independent replicate. Each data point represents an independent experimental replicate (*n* = 4) and the line represents the average. Two-way ANOVA with Dunnett’s test for multiple comparisons was used to determine statistical significance (*p < 0.05, **p < 0.01, ***p < 0.001, ****p < 0.0001)

Protein expression profiles obtained from the proteomics dataset were analyzed using the Ingenuity Pathway Analysis (IPA) bioinformatics platform (Qiagen) to identify inhibitors of overrepresented signaling pathways. Comparative analyses of log2 expression values of the proteins that were differentially expressed in response to IL-17A/F + TNF-α (Supplementary Table I), identified PI3K inhibitor LY294002, PKC inhibitor GO6976, and MEK inhibitor PD98059, as upstream chemical inhibitors for proteins that were significantly altered by IL-17A/F + TNF-α compared to either cytokine alone. Based on these *in silico* results, HBEC-3KT cells were pre-treated with LY294002, GO6976 and PD98059 at various concentrations (4 to 16 µM) for one hour at 37ºC, prior to stimulation with IL-17A/F, TNF-α, or IL-17A/F + TNF-α cytomix as indicated. TC supernatants collected 24 h after stimulation were used to examine the protein abundance of LCN-2, Elafin, GROα, and IL-8, as these proteins were demonstrated to be synergistically enhanced by the combination of IL-17A/F and TNF-α (Fig. 3). PI3K inhibitor LY294002 significantly suppressed IL-17A/F + TNF-α-mediated production of LCN-2 and Elafin at all concentrations tested, in a concentration-dependent manner (Fig. 5 A). PKC inhibitor GO6976 also decreased the production of LCN-2 and Elafin, albeit at the higher concentrations (Fig. 5B). MEK inhibitor PD98059 suppressed Elafin production in a dose dependent manner but did not affect LCN-2 production (Fig. 5 C). In contrast, none of the inhibitors suppressed IL-17A/F + TNF-α-mediated production of neutrophil chemokines GROα and IL-8 (Supplemental Fig. 4). These results demonstrated that PI3K and PKC pathways selectively controlled the synergistic effect of IL-17A/F + TNF-α-mediated production of HDPs, LCN-2 and Elafin, but not the production of IL-8 and GROα, in bronchial epithelial cells. These results suggested that disparate mechanisms are involved in the synergistic enhancement of HDPs and chemokines, mediated by the combination of IL-17A/F and TNF-α.

### The combination of IL-17A/F and TNF-α uniquely enhances neutrophil migration

**Fig. 6:**
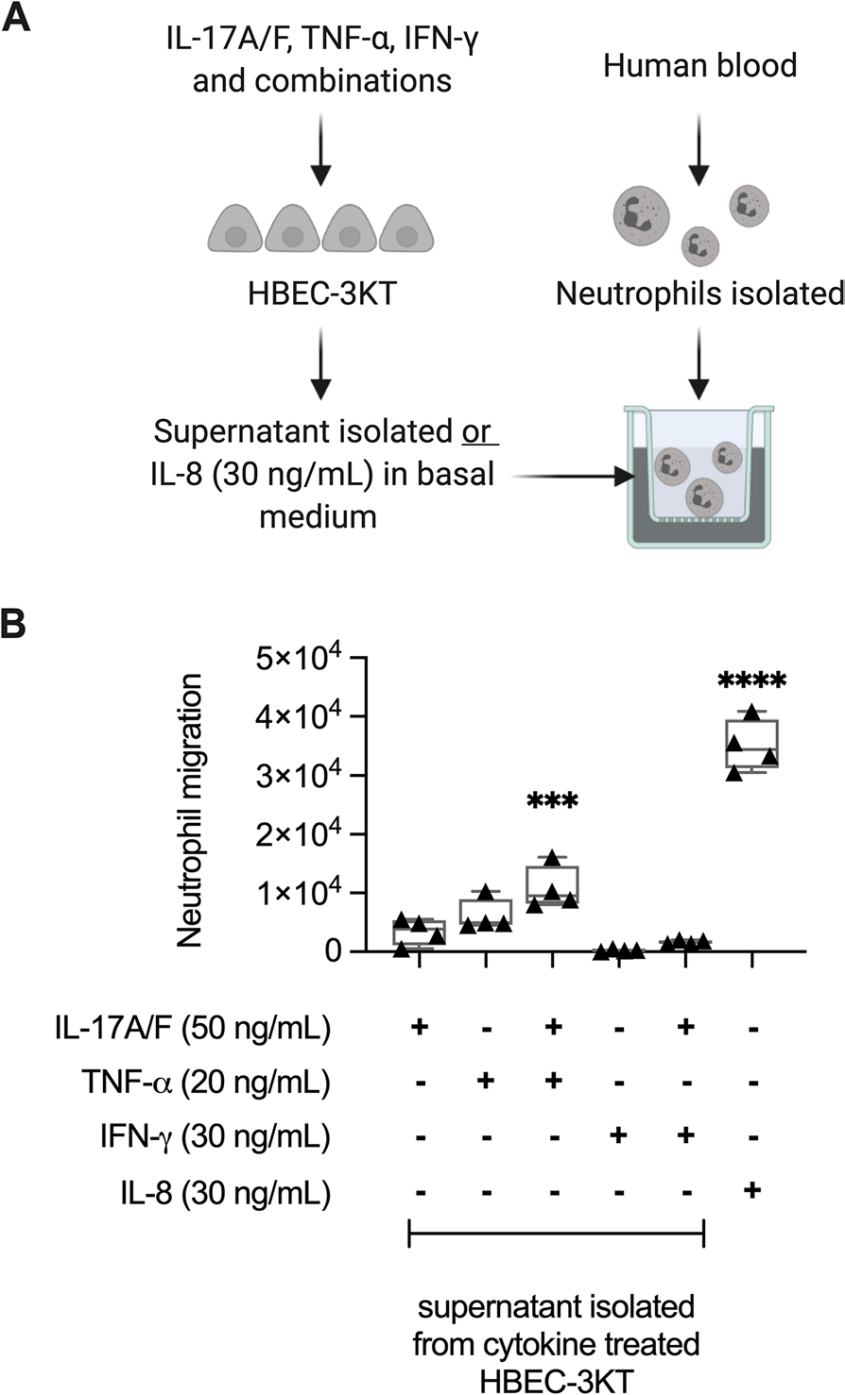
Functional validation of neutrophil migration enhanced by the combination of IL-17A/F and TNF-α. (**A**) HBEC-3KT were stimulated with IL-17A/F (50 ng/mL), TNF-α (20 ng/mL) and IFN-γ (30 ng/mL), or the cytomix combinations, as indicated. Tissue culture supernatants were collected after 24 h and used in trans-well cell migration assays, to monitor the migration of neutrophils isolated from human blood. Cell culture medium spiked with human recombinant IL-8 (30 ng/mL) was used as a positive control. (**B**) Results are shown as boxplots with the median line and IQR, and whiskers show minimum and maximum values. Each data point represents an independent experimental replicate with HBEC supernatant (*n* = 4), using neutrophil isolated from one donor. Each dot represents the average number of neutrophils that traversed the membrane within two hours in each experiment. Increase in neutrophil migration was calculated after subtraction of neutrophil numbers found with tissue culture supernatant from paired unstimulated cells in trans-well migration assay, for each biological replicate. One-way ANOVA with Bonferroni’s post-hoc test for multiple comparisons was used for statistical significance compared to unstimulated cells as control (***p < 0.001, ****p < 0.0001)

Chemokines IL-8 and GROα, as well as HDPs LCN-2 and Elafin, are known to contribute to neutrophil accumulation at sites of inflammation [27–30]. Chemoattractant functions of these proteins are primarily mediated once secreted, and our results demonstrated that combination of IL-17A/F and TNF-α synergistically enhances the abundance of these proteins secreted from human bronchial epithelial cells, compared to either cytokine alone (Fig. 3). These results suggested that the combination of IL-17A/F and TNF-α may synergistically enhance neutrophil migration. Therefore, we further aimed to functionally validate the effect of the combination of IL-17A/F and TNF-α on neutrophil migration. HBEC-3KT cells were stimulated with IL-17A/F (50 ng/mL) in the presence and absence of TNF-α (20 ng/mL) for 24 h. Subsequently TC supernatants were used in the bottom chamber of Transwell plates to examine trans-well migration of neutrophils isolated from human blood (Fig. 6 A). Recombinant chemokine IL-8 (30 ng/mL) was used as a positive control. TC supernatant obtained from cells stimulated with the combination of IL-17A/F and TNF-α significantly enhanced neutrophil migration, compared to that obtained from unstimulated cells (Fig. 6B). TC supernatants obtained from cells stimulated with either IL-17A/F or TNF-α alone did not significantly enhance neutrophil migration (Fig. 6B). These results indicated that factors secreted in the TC supernatants obtained from bronchial epithelial cells stimulated with the combination of IL-17A/F and TNF-α uniquely enhanced neutrophil migration.

To further examine the involvement of PI3K and PKC pathways on IL-17A/F + TNF-α mediated enhancement of neutrophil migration, TC supernatant from HBEC-3KT stimulated with the combination of IL-17A/F (50 ng/mL) and TNF-α (20 ng/mL), in the presence and absence of pharmacological inhibitors LY294002 (PI3Ki; 16 µM) and GO6976 (PKCi; 16 µM), were used in the bottom chamber of Transwell plates to examine trans-well migration of human neutrophils as discussed above. Presence of the pharmacological inhibitors (16 µM) suppressed the production of LCN-2 and Elafin (Supplementary Fig. 5 A), consistent with results shown in Fig. 6. However, enhanced neutrophil migration mediated by the combination of IL-17A/F and TNF-α was not altered in the presence of pharmacological inhibitors of PI3K and PKC pathways (Supplementary Fig. 5B).

### In vivo confirmation of selected protein targets in a murine model of airway inflammation

**Fig. 7:**
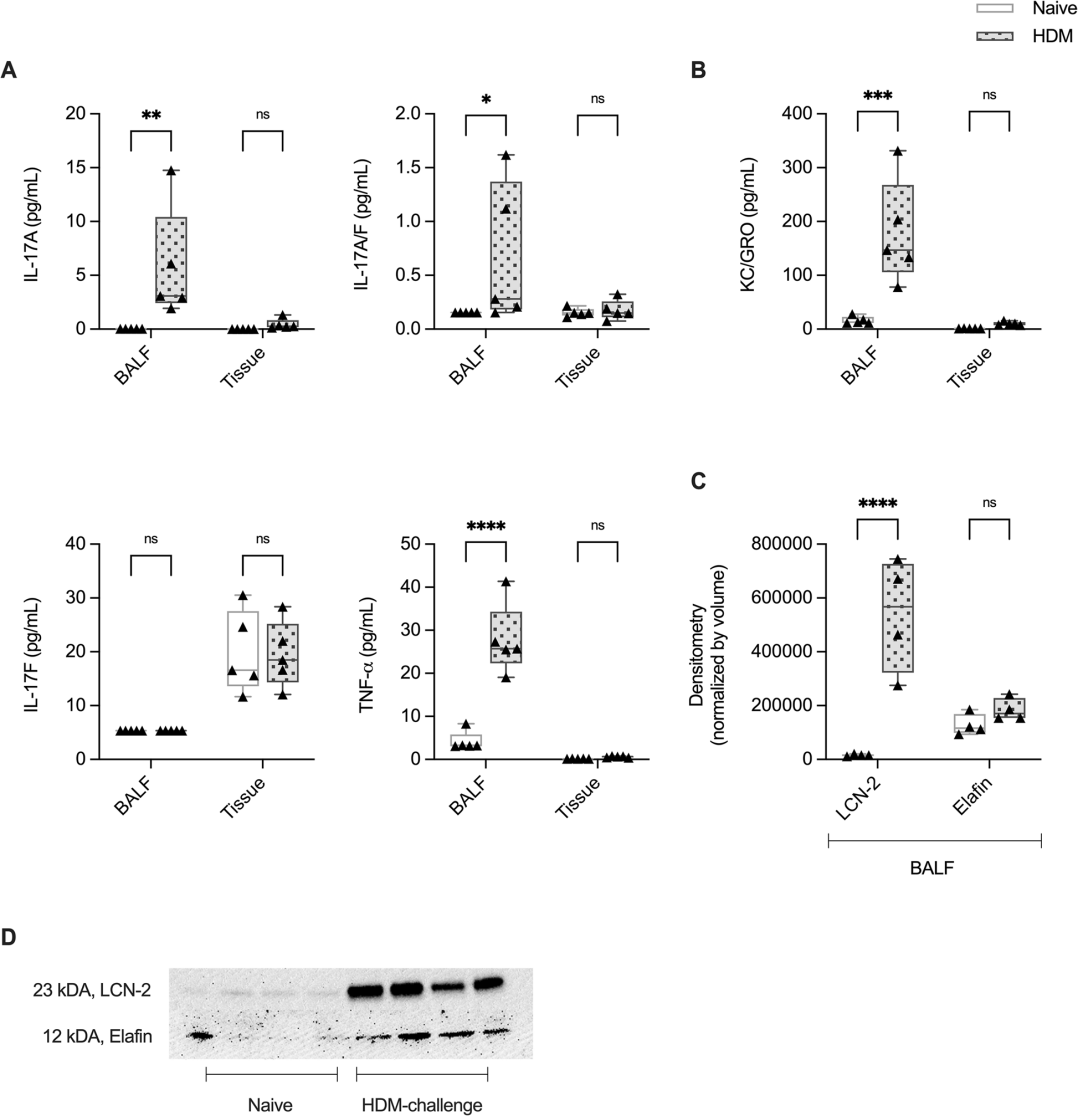
Assessment of protein production in the lungs of a murine model of HDM-induced airway inflammation. 8-10-week-old female BALB/C mice were challenged by intranasal administration of 35 µL of whole HDM extract (0.7 mg/mL) in saline for two weeks. BALF and lung tissue lysates obtained from allergen-naïve (*n* = 5) and HDM-challenged (*n* = 5) mice were monitored for the abundance of (**A**) cytokines IL-17A, IL-17A/F, IL-17F and TNF-α, and (**B**) KC by multiplex Meso Scale Discovery (MSD) platform. Undetectable values of cytokine were assigned a value of 1/4 the minimum detectable limit. (**C**) BALF of HDM-challenged (*n* = 4) and allergen-naïve (*n* = 4) mice were monitored for the abundance of LCN-2 and Elafin by Western blot. (**D**) Representative blot for LCN-2 and Elafin. Results are shown as boxplots, wherein bars show median and IQR, and whiskers show minimum and maximum points. Each data point represents an individual mouse. Statistical analysis was performed using two-way ANOVA with Bonferroni’s post-hoc test for multiple comparisons. Statistical significance denotes differences compared to control (*p < 0.05, **p < 0.01, ***p < 0.001)

We have previously shown that intranasal challenge of mice for two weeks with house dust mite (HDM) results in airway inflammation and hyperresponsiveness, along with elevated abundance of neutrophils in the lungs, 24 h after the last HDM challenge [26, 33, 34]. To corroborate our in vitro findings in a physiologically representative model of airway inflammation, we measured IL-17A, IL-17F, heterodimer IL-17A/F and TNF-α in bronchoalveolar lavage fluid (BALF) and lung tissue lysates from mice challenged with HDM for two weeks. IL-17A and IL-17A/F, but not IL-17F, were significantly higher in BALF from HDM-challenged mice, compared to allergen-naïve mice (Fig. 7 A). The concentration of TNF-α was also significantly higher in the BALF from HDM-challenged mice (Fig. 7 A). We next examined the abundance of HDP LCN-2, Elafin, and the murine neutrophil chemokine KC (mouse homolog of human GROα) in BALF and lung tissue lysates. Abundance of LCN-2 and KC was significantly increased in BALF, but not in the lung tissue lysates, from HDM-challenged mice (Fig. 7B C). These results demonstrated a concurrent increase of IL-17A/F and TNF-α in BALF of allergen-challenged mice, along with the neutrophil chemoattractant proteins targets that we had identified to be synergistically enhanced by IL-17A/F and TNF-α in human bronchial epithelial cells (Figs. 3 and 4).

## Discussion

In this study, we showed that the combinatorial effect of IL-17A/F and TNF-α uniquely alters the proteome of human bronchial epithelial cells (HBEC), and enhances proteins in primarily three functional categories, neutrophilic chemokines, HDPs with antimicrobial and immunomodulatory functions, and airway remodeling factors. In independent confirmatory studies, we demonstrated that the combination of IL-17A/F and TNF-α synergistically enhances the production of LCN-2, Elafin, GROα and IL-8, in TC supernatants from HBEC. Interestingly, the two HDPs (LCN-2 and Elafin) identified to be synergistically enhanced by IL-17A/F and TNF-α also promote neutrophil migration [27, 28, 30]. These findings were functionally corroborated by our results demonstrating that secreted factors from HBEC stimulated with the combination of IL-17A/F and TNF-α uniquely promote neutrophil migration, while those from cells stimulated with either cytokine alone do not. In further mechanistic studies, we showed that PI3K and PKC pathways are involved in the synergistic enhancement of HDPs LCN-2 and Elafin, but not neutrophilic chemokines. Our results indicate that disparate pathways control the synergistic enhancement of the HDPs compared to the induction of chemokines, in response to concurrent activation by IL-17A/F and TNF-α in HBEC. We also demonstrated in vivo that IL-17A/F and TNF-α, as well as the proteins identified from the in vitro studies i.e. LCN-2 and the mouse homolog of GROα (KC), are all significantly increased in the BALF of allergen-challenged mice, using a model of airway inflammation known to increase neutrophil accumulation in the lungs [26, 33, 34]. These results are corroborated by previous studies demonstrating individual HDP and chemokine induction in response to IL-17 [28, 35]. Overall, the findings in this study identify proteins that are uniquely altered in response to concurrent activation with the heterodimer IL-17A/F and TNF-α in the lung, and demonstrate that the synergistic effect of these two cytokines leads to the enhancement of secreted proteins that are known to promote neutrophil migration in the context of airway inflammation.

To our knowledge, this is the first study to detail proteins that are uniquely or synergistically altered by the combined effect of IL-17A/F and TNF-α in HBEC, using a proteomics approach. Although previous studies have demonstrated synergy between IL-17 and TNF-α in promoting inflammatory responses in different cell types such as endothelial cells, hepatocytes, synovial fibroblasts and human airway epithelial cells, these studies were primarily focused on IL-17A [18, 36–39]. Relative quantitation of protein candidates identified to be uniquely or synergistically enhanced by the combination of IL-17A/F and TNF-α within the bronchial epithelial proteome in this study, suggests that the IL-17A/F-centric protein biosignature is further enhanced by TNF-α, with the concurrent activation with these two cytokines. This is corroborated by a recent study demonstrating that pro-inflammatory responses mediated by IL-17A and IL-17F are potentiated by TNF-α in synoviocytes [40]. A previous study had demonstrated that IL-17A enhances the expression CXCL3, CSF3, SAA1 and CCL20 in primary airway epithelial cells [8], and these molecular candidates were also found to be enhanced by the combination of IL-17A/F and TNF-α in our proteomics dataset. It is thus likely that the combination of IL-17A and TNF-α may result in a similar protein biosignature that is defined in this study using the heterodimer IL-17A/F. Nonetheless, taken together these studies indicate that acute pro-inflammatory cytokines such as TNF-α, produced in the presence of pathogenic and/or environmental factors e.g., air pollution, allergens and fungi, can exacerbate IL17A/F-mediated responses to promote airway inflammation [4, 41, 42].

The pathophysiology of chronic respiratory diseases characterized by airway inflammation is known to be driven by the cooperative interaction between various pro-inflammatory mediators [14]. TNF-α along with IL-17 family of cytokines, including IL-17A/F, is enhanced in severe asthma [1–8, 20, 23–25]. Asthma is a heterogenous respiratory disease wherein based on the accumulation of leukocytes in the lungs it can be broadly classified into four prominent endotypes; eosinophilic, neutrophilic, mixed eosinophilic/neutrophilic and paucigranulocytic (no cellular accumulation in the lungs) [43]. Typically eosinophilic asthma results in elevated levels of T helper (Th) 2 cytokines such as IL-4, IL-13 and IL-5, along with eotaxins, which drive eosinophil accumulation in the lungs [44]. Gene expression profiling in endobronchial tissues in patients with treatment-refractory severe asthma has shown that Th2-high and Th17-high gene expression profiles are mutually exclusive [45]. Furthermore, neutralization of IL-4 and IL-13 can result in increased Th17 cells in the lungs [45]. Based on these previous studies, it may be speculated that the concurrent Th17/Th1 driven neutrophil-skewed inflammation and Th2-driven eosinophilic inflammation in the lungs may be reciprocally regulated. In general, neutrophilic inflammation represents a non-Th2 disease with elevated levels of cytokines such as IL-17A/F and TNF-α in the lungs. A previous study showed that IL-8 was significantly elevated in the lungs of severe asthma patients, and that the levels of IL-8 associated with the abundance of neutrophil elastase, a marker for neutrophil activation [46]. Here, we demonstrate that the combined activity of IL-17A/F and TNF-α synergistically enhances neutrophil chemoattractants such as IL-8 and LCN-2. We also show that the combination of IL-17A/F and TNF-α is required for neutrophil migration compared to either cytokine alone. Thus, taken together it may be speculated that the combinatorial effect of IL-17A/F and TNF-α may be a key contributing factor in facilitating neutrophilic inflammation in the lungs in severe asthma.

Research in the phenotypic heterogeneity of asthma has shown that the immunophenotype of severe asthma is complex, which includes both Th2-high and Th2-low/Th1 + Th17-high disease [1, 2]. Typically, severe steroid-unresponsive asthma characterized by neutrophilia exhibits a Th2-low and Th17-high airway inflammation, with elevated levels of IL-17A, IL-17A/F and TNF-α at mucosal surfaces of the airway [1, 2, 47–49]. Neutrophil accumulation in the lung results in Neutrophil-Extracellular Trap (NET) formation in the airways [1, 50], which can further increase Th17 differentiation and subsequently IL-17A/F production [1]. In addition, neutrophils are also capable of recruiting Th17 cells via CCL20 and CCL2 [51]. Therefore, neutrophilic accumulation in the airways may prolong IL-17A/F-mediated airway inflammation through a positive feedback loop, resulting in sustained inflammation and subsequent tissue damage. The only IL-17 family member produced by airway epithelial cells is IL-17C, which is released by epithelia following activation with various stimuli, including pro-inflammatory cytokines such as TNF-α [52]. IL-17C enhances the transcription of downstream targets which includes S100A9, GROα, IL-8, CSF3 and CCL20 via the activation of IL-17RA and IL-17RE receptors [53]. Although, we did not show the involvement of autocrine IL-17C signaling in the combinatorial effect of IL-17A/F and TNF-α, some of the IL-17C targets such as GROα and IL-8 were shown to be synergistically enhanced by the combination of IL-17A/F and TNF-α in this study. Therefore, it is possible that concurrent activation with IL17A/F and TNF-α may enhance IL-17C abundance in bronchial epithelial cells, perhaps at an earlier time point than that examined in this study, thus amplifying the autocrine activity of IL-17C. Interestingly, clinical trials with either anti-TNF-α strategies or blocking the IL-17RA receptor did not adequately control severe asthma [54–56]. In this context, the list of proteins and pathways defined in this study may be valuable to design new interventions to specifically target the combinatorial effect of IL-17A/F and TNF-α for the control of severe asthma.

Molecular mechanisms that underlie the cooperative effect of IL-17A/F and TNF-α are not completely understood. IL-17A, IL-17F and IL-17A/F signal via the heterodimeric receptor IL-17RA/RC, with varying affinities [57]. These IL-17 members induce modest levels of downstream signaling and inflammatory responses, instead synergistically enhance signaling pathways through cooperative effect with acute pro-inflammatory cytokine such as TNF-α [58]. Previous studies have demonstrated that synergistic effects of IL-17A and TNF-α are mediated through the activation of pathways such as NF-κB, ERK mitogen-activated protein kinase (MAPK), protein kinase B and PI3K pathways [36, 59, 60]. Aligned with this, here we demonstrate that the synergistic effect of IL-17A/F and TNF-α involves the PI3K and PKC pathways in HBEC. However, our results also suggest that there may be disparate signaling mechanisms that control different downstream responses mediated by the combinatorial action of IL-17A/F and TNF-α. This is indicated by our results demonstrating that cytomix IL-17A/F + TNF-α-mediated synergistic enhancement of HDPs LCN-2 and Elafin is dependent on PKC and PI3K signaling pathways, but not the enhancement of chemokines IL-8 and GROα. Mechanisms previously suggested for the synergistic effects of IL-17A and TNF-α include IL-17A-mediated increase in the expression of TNF-α receptor II in hepatocytes and synoviocytes [36, 61], and post-transcriptional mRNA stabilization of TNF-α-induced chemokines by IL-17A [62–64]. As TNF-α-family receptors were not demonstrated to be uniquely enhanced by the combination of IL-17A/F and TNF-α in our proteomics dataset, our results suggest that the combinatorial effects of IL-17A/F and TNF-α may not be driven by TNF-α-receptor. Previous studies have shown that TNF-α results in increased abundance of mRNA and protein production of the neutrophilic chemokines through activation of the NF-κB pathway, while IL-17A drives chemokine mRNA stabilization through an Act-1 dependent mechanism [62, 63]. However, chemokines defined in this study, Groα and IL-8, were enhanced in a supra-additive manner both at the transcriptional level and protein production. Thus, our results suggest that the mechanisms associated with the synergistic enhancement of neutrophilic chemokines such as Groα and IL-8, by the concurrent action of IL-17A/F and TNF-α, is not solely dependent on post-transcriptional regulation.

It is likely that there may be indirect effects through the concurrent actions of IL-17A/F and TNF-α, for example on Elafin production. A possible mechanism is that the combination of IL-17A/F and TNF-α enhances post-transcriptional machinery that aids in the conversion of constitutively expressed Elafin mRNA into newly formed mature protein. This is corroborated by previous studies demonstrating that the combination of TNF-α and endotoxin enhances the production and secretion of Elafin in the absence of upregulation of mRNA expression [27, 65, 66]. Another possibility of indirect influence of IL-17A/F and TNF-α may be on protein processing of Elafin. Antimicrobial HDPs such as Elafin are often constitutively expressed as precursor proteins that are rapidly cleaved and released as mature peptides in response to pathogenic and inflammatory stimuli [67]. Therefore, it is possible that the combination of IL-17A/F and TNF-α may mediate changes in yet unidentified post-transcriptional or translational machinery to enhance the production of proteins such as Elafin in bronchial epithelial cells, without influencing mRNA abundance.

Our findings highlight the complex and overlapping signaling mechanisms involved in the regulation of downstream responses and functional outcomes mediated by the concurrent activation of IL-17 family of cytokines and TNF-α in the lungs. For example, we demonstrate that although the production of LCN-2 and Elafin are dependent on PI3K and PKC pathways, neutrophil migration mediated by the combinatorial action of IL-17A/F and TNF-α is not. This shows that the concurrent presence of IL-17A/F and TNF-α enhances secreted proteins with redundant functions in the context of neutrophil recruitment. It is likely, that enhancement of GROα by the combinatorial effect of IL-17A/F and TNF-α, along with the enhancement of other functional analogues of GROα such as GROβ (*CXCL2*) and GRO3 (*CXCL3*), as identified from our proteomics profiling [68], maintain neutrophil migration in the absence of LCN-2 and Elafin. These findings also suggest although LCN-2 and Elafin are known to promote neutrophil migration and activation [30, 69] they may be dispensable in facilitating neutrophilia. Overall, our findings highlight the complexity of the interplay of IL-17A/F and TNF-α in the context of neutrophil migration, and the functional redundancy of target proteins that are synergistically enhanced by the concurrent actions of IL-17A/F and TNF-α, which warrants further investigation.

A limitation of this study is that the protein targets defined were at a single time point, and only in submerged bronchial epithelial cells stimulated with IL-17A/F, TNF-α and their combinations. In addition, in vivo results reported in this study were from one HDM-challenged mouse model. Also, this study only focused on proteins that were identified to be enhanced by the combinatorial effects of IL-17A/F and TNF-α in independent validation experiments. Further examination of protein targets that were identified to be suppressed in response to the combined activity of IL-17A/F and TNF-α could provide valuable mechanistic data for delineating molecular processes related to the pathophysiology of neutrophilic airway inflammation and severe asthma. Nevertheless, the results of this study provide the foundation to further investigate the complex interplay of IL-17A/F and TNF-α in different mouse models and physiologically representative mucocilliary-differentiated bronchial epithelial cell culture systems. Future studies using air-liquid interface (ALI) bronchial epithelial cell culture, as well as chronic and recall models of allergen challenge, using different clinically relevant allergens, will provide an insight into the complex interplay of cytokines elevated in the lungs and how their interplay with IL-17A/F and TNF-α facilitates chronic neutrophilic inflammation.

## Conclusion

In summary, the findings in this study provide insight into the fundamental understanding of downstream protein targets and pathways in response to the combinatorial activity of TNF-α along with the IL-17-family heterodimer IL-17A/F, in the context of airway inflammation. The protein targets identified in this study will be useful for the development of interventional strategies to target biological processes enhanced by the concurrent presence of IL-17A/F and TNF-α, relevant to chronic respiratory diseases such as steroid-unresponsive severe asthma.

## Methods

### Epithelial cell isolation and culture

HBEC-3KT cell line was obtained from American Type Culture Collection (ATCC® CRL-4051™). These cells were cultured in airway epithelial cell basal medium (ATCC® PCS-300-030™) and supplemented with bronchial epithelial cell growth kit (ATCC® PCS-300-040™), according to the manufacturer’s instructions. HBEC-3KT were maintained at ~ 80% confluency, trypsinized with 1:3 dilution of 0.5% trypsin-EDTA (Invitrogen™, Life Technologies Inc, Burlington, ON, Canada) in PBS. Culture medium was changed to airway epithelial cells basal medium containing 6 mM L-glutamine without growth factors, 24 h prior to stimulation with various cytokines as indicated.

Human PBEC were isolated from resected tumor-free lung tissues obtained from four anonymized donors (*n* = 4) undergoing lung resection surgery for lung cancer at the Leiden University Medical Centre (The Netherlands), as previously described [26, 70]. Use of such lung tissue that became available for research within the framework of patient care was in line with the “Human Tissue and Medical Research: Code of conduct for responsible use” (2011) (www.federa.org), that describes the opt-out system for coded anonymous further use of such tissue. PBEC were expanded in T75 flasks pre-coated with coating media (containing 30 µg/mL PureCol (Advanced Biomatrix, California, USA), 10 µg/mL fibronectin (Sigma), 10 µg/mL bovine serum albumin (BSA; Sigma) in PBS (Gibco)), and maintained in supplemented keratinocyte serum-free medium (KSFM; Gibco) containing 0.2 ng/mL epidermal growth factor (EGF; Life Technologies), 25 µg/mL bovine pituitary extract (BPE; Gibco), 1 µM isoproterenol (Sigma) and 1:100 dilution of antibiotics Penicillin and Streptomycin (Lonza), until ~ 80% confluent. PBEC were trypsinized with 0.3 mg/mL trypsin (Gibco) containing 0.1 mg/mL EDTA (Gibco), 1 mg/mL glucose (Gibco) and 1:100 dilution of Penicillin and Streptomycin, in PBS. PBEC were seeded at a density of 5000/cm^2^ in TC plates pre-coated with coating media (as described above). PBEC were cultured with a 1:1 mixture of supplemented Dulbecco’s modified Eagle’s medium (DMEM; Gibco) with a 1:40 dilution of HEPES (Invitrogen), and basal bronchial epithelial cell medium (ScienCell) containing bronchial epithelial cell growth supplement (ScienCell), a 1:100 dilution of Penicillin/Streptomycin and 1 nM of a light stable analog of retinoic acid, EC-23 (Tocris, UK). PBEC were cultured to a maximum of ~ 80% confluency with the culture medium replaced every 48 h. Culture medium was replaced 24 h prior to stimulation with various cytokines with medium without EGF, BPE, BSA and hydrocortisone (starvation media).

### Cytokines, inhibitors, and antibody reagents

Recombinant human cytokines IL-17A/F (carrier-free), TNF-α and IFNγ were all obtained from R&D Systems (Oakville, ON, CA). Pharmacological inhibitors, phosphoinositide 3-kinase (PI3K) inhibitor LY294002, protein kinase-C (PKC) inhibitor GO6976 and MAPK/ERK kinase (MEK) inhibitor PD98059 were obtained from SelleckChem (Burlington, ON, CA). The inhibitors were used at a concentration range according to the manufacturer’s instructions. HBEC-3KT cells were pre-treated with the selected inhibitors reconstituted in DMSO (then diluted in airway epithelial cells basal medium containing 6 mM L-glutamine without growth factors to a final dilution of < 1:2000 v/v) one hour prior to cytokine stimulation. Antibodies specific to anti-mouse LCN-2, IL-17A/F and TNF-α, and anti-human Elafin were all obtained from Abcam (Toronto, ON, Canada). Anti-human actin antibody was obtained from Millipore (Burlington, MA, USA). HRP-linked purified anti-rabbit IgG, anti-goat IgG, and anti-mouse IgG-secondary antibodies were all obtained from Cell Signaling Technology (distributed by New England Biolabs, ON, Canada).

### Slow off-rate modified aptamer-based proteomic array

HBEC-3KT were stimulated with IL-17A/F (50 ng/mL), TNF-α (20 ng/mL) or IFN-γ (30 ng/mL) for 24 h. Cytokine concentrations and time points were selected based on our previous studies [21, 26]. Total cell lysates were prepared in lysis buffer containing M-PER™ (ThermoFisher Scientific, Burlington, ON, Canada) and HALT protease and phosphatase inhibitor cocktail (ThermoFisher Scientific). Protein concentration was determined by microBCA protein assay kit (Thermo Fisher Scientific, Massachusetts, USA). 14 µg total protein per sample obtained from five independent experiments were probed independently using the Slow off-rate Modified Aptamer (SOMAmer^®^)-based proteomic array (SomaLogics®-licensed platform at the Manitoba Center of Proteomics and Systems Biology, Canada) as previously described by us [21]. This technology uses high affinity binding aptamer-based probes called SOMAmers™ (SomaLogic Inc., Boulder CO, USA), with each aptamer (single strand oligonucleotide that bind to protein) probe binding to a specific human protein. The SOMAmer® V.2 protein arrays were used for profiling the abundance of 1322 protein targets in each sample. The arrays were processed and analyzed according to the manufacturer’s recommended protocol (SOMALogic, Inc) and as detailed in previous studies [21, 71–74]. Protein abundance was quantified using the Agilent hybridization array scanner in relative fluorescence unit (RFU), as previously described [21, 71–74]. The RFU readout values were log2-transformed and used for pairwise differential analysis using an uncorrected Welch’s T-test. Proteins that were significantly different in abundance (≥2-fold, *p* < 0.05) in response to IL-17A/F and TNF-α cytomix condition, compared to either cytokine alone, were selected for further analyses. Heatmap with hierarchical clustering was generated using the Multi-Experiment Viewer Version 10.2 and GraphPad PRISM 9 was used for visual representation of changes in protein expression profile.

Proteins that were significantly altered by the cytomix IL-17A/F and TNF-α, compared to either cytokine alone, were used for further analyses using the IPA bioinformatics software (Qiagen), to predict overrepresented pathways and associated chemical inhibitors. In addition, statistically significant pathway enrichment was determined by selecting proteins that were significantly enhanced by the combination of the two cytokines compared to each cytokine alone using an in-house analytical tool, which was developed to compute enrichment specific to the SOMAmer®-based collection of > 1300 proteins. An enrichment score was used for this analysis which represented the probability that the submitted collection of proteins would occur within a given biological process due to randomness.

### Quantitative real-time PCR (qRT-PCR)

HBEC-3KT cells were stimulated with cytokines as indicated for 6 h and total RNA isolated using the Qiagen RNAeasy Plus Mini Kit according to the manufacturer’s instructions. Total RNA was eluted in RNAse-free water (Ambion). RNA concentration and purity were determined using a NanoDrop 2000 Spectrophotometer (ThermoFisher Scientific). mRNA expression was analyzed using SuperScript III Platinum Two-Step qRT-PCR Kit with SYBR Green (Invitrogen), according to the manufacturer’s instructions, in the ABI Prism 7000 sequence detection system (Applied Biosystems, CA, USA), and as previously described by us [75]. Briefly, 100 ng of total RNA was reverse transcribed in a 20 µl reaction volume for 10 min at 25 °C, followed by 50 min at 42 °C, after which the reaction was stopped by incubating the reaction solution at 85 °C for 5 min. cDNA was aliquoted and stored at -20 °C until used. For qRT-PCR amplification, a reaction mix containing 2.5 µL of 1/10 diluted cDNA template, 0.5 µL of 10 µM primer mix, 6.25 µL of Platinum SyBr Green qPCR-Super-Mix UDG with Rox reference, making up the total volume to 12.5 µL with RNase-free water was used. Primers used for qRT-PCR are detailed in Table 1. PCR specificity was measured by melting curve analysis. Fold changes were calculated using the comparative ΔΔCt method [76], after normalization with 18 S RNA, which was unchanged in response to pro-inflammatory cytokines (data not shown).

**Table 1:** Primers used for quantitative real-time PCR

Gene	Forward Primer (5’-3’)	Reverse Primer (5’-3’)
*NGAL2 (LCN-2)*	CTCCACCTCAGACCTGATCC	ACATACCACTTCCCCTGGAAT
*IL-8*	AGACAGCAGAGCACACAAGC	AGGAAGGCTGCCAAGAGAG
*CXCL1* (GROα)	TCCTGCATCCCCCATAGTTA	CTTCAGGAACAGCCACCAGT
*PI3* (Elafin)	TTATCCCTTGTAAATACCACAGACC	GCCATACCAATCTTTATGCAGTC
*MMP-13*	CCAGTCTCCGAGGAGAAACA	AAAAACAGCTCCGCATCAAC
*18 S RNA*	GTAACCCGTTGAACCCCATT	CCATCCAATCGGTAGTAGCG

### ELISA

 TC supernatants were centrifuged at 250×g for 5 min to obtain cell-free samples and the aliquots were stored at -20ºC until use. Abundance of HDP (LCN-2 and Elafin), chemokines (GROα and IL-8), and MMP13 were measured in the TC supernatants by ELISA using specific antibody pairs (R&D Systems), as per the manufacturer’s instructions. Production of the chemokine MCP-1 was monitored using an ELISA kit obtained from eBioscience/ThermoFisher Scientific (Mississauga, ON, CA), as per the manufacturer’s instructions.

### Western blots

 Cells were washed with cold PBS, scraped from 60 mm TC plates using a 25 cm cell scrapper (VWR) and collected in phosphate-buffered saline (PBS) containing protease inhibitor cocktail (Cell Signaling Technology, Massachusetts, USA). Cells were centrifuged at 250×g for 5 min. The cell pellets were lysed in PBS containing Protease Inhibitor Cocktail (PIC) (New England Biolabs, ON, Canada) and 0.5% NP40 (Sigma, Missouri, USA). Cell pellets underwent one 24 h freeze thaw cycle prior to centrifuging at 10 000×g for 10 min to obtain cell-free lysates. Total protein concentration was determined using a microBCA protein assay kit (Thermo Fisher Scientific, Massachusetts, USA). Equal amounts of protein (10 µg) were resolved on 4–12% NuPage^™^ 10% Bis-Tris Gels (Invitrogen) followed by transfer to nitrocellulose membranes (Millipore, Massachusetts, USA). Membranes were blocked with Tris-buffered saline (TBST) (20 mM Tris–HCl, pH 7.5, 150 mM NaCl, 0.1% Tween-20) containing 5% milk powder. Membranes were probed for antibodies (as indicated above) in TBST containing 2.5% milk powder then developed using ECL Prime detection system (Thermo Fisher Scientific, Massachusetts, USA) according to the manufacturer’s instructions.

### Neutrophil isolation and migration assay

 Venous blood was collected in EDTA vacutainer tubes, from healthy volunteers with written informed consent, according to a protocol approved by the University of Manitoba Research Ethics Board. Human neutrophils were isolated using EasySep™ Direct Human Neutrophil Isolation Kit (STEMCELL technologies Canada Inc., Vancouver, BC, Canada) according to the manufacturer’s protocol. Briefly, ~ 25 ml of blood was mixed gently with the isolation cocktail and 50 µL of RapidSpheres™ provided in the kit and incubated for 5 min at room temperature (RT). D-PBS (containing 1 mM EDTA and free of Ca2 + and Mg2+) was added to make up the total volume to 50 mL, mixed gently, and neutrophils were isolated through magnetic negative selection for 10 min. The clear cell suspension was once again subjected to magnetic separation using RapidSpheres™ according to the manufacturer’s instructions, to obtain enriched human neutrophils.

TC supernatants were collected from HBEC-3KT cells stimulated with IL-17A/F (50 ng/mL) or TNF-α (20 ng/mL) or IFN-γ (30 ng/mL), or cytomix as indicated, for 24 h. TC supernatants (600 µL) were added to the bottom chamber of a Transwell plate. The plates were incubated at 37ºC in a humidified chamber with 5% of CO2 for 30 min. Neutrophils isolated from human blood (6 × 10^5^ cells/well, 200 µL) were added to the upper chamber of the inserts of 5.0 µM polycarbonate membrane Transwell permeable supports (Costar, Corning, NY, USA) and incubated for 2 h. The number of neutrophils that migrated to the bottom chamber was counted using a Scepter™ 2.0 Handheld Automated Cell Counter (Millipore Ltd, ON, Canada). Human recombinant neutrophil chemokine IL-8 (30 ng/mL) in airway epithelial cells basal medium (containing 6 mM L-glutamine) was used in the bottom chamber as a positive control [75]. Previous studies have shown that IL-8 concentration in tracheal aspirates of patients with acute severe asthma can be as high as 75 ng/mL [77]. Thus, IL-8 used as a positive control was within the physiological range of concentration relevant to this study. In addition, HBEC-3KT cells were stimulated with combination of IL-17A/F (50 ng/mL) and TNF-α (20 ng/mL) in the presence and absence of pharmacological inhibitors LY294002 (PI3Ki; 16 μm) and GO6976 (PKCi; 16 μm) as indicated. TC supernatants collected after 24 h stimulation were examined for the abundance of LCN-2 and Elafin by ELISA, and used in bottom chamber of trans-well plates to examine the migration of neutrophils isolated from human blood, as mentioned above.

### Mouse model of house dust mite-challenged airway inflammation

 HDM-challenge protocol used in this study was previously described by us [26, 33], approved by the University of Manitoba Animal Research Ethics Board, and compliant with ARRIVE guidelines for in vivo animal research [78]. We have previously shown that repeated HDM challenge for two weeks results in airway inflammation and hyperresponsiveness, along with significant neutrophil and eosinophil accumulation in the lungs, differential expression of a network of genes related to allergy and asthma, and enhanced levels of cytokines such as IL-17A, 24 h after the last HDM challenge [26, 33, 34]. These previous studies clearly indicate a mixed eosinophil and neutrophilic inflammatory profile following two weeks of repeated HDM instillations when outcomes are examined 24 h after the last HDM challenge. Thus, based on these previous studies, here we used repeated instillation of HDM for two weeks and monitored cell accumulation and protein production in the lungs 24 h after the last HDM challenge. Briefly, female BALB/c mice (6 to 7 weeks) were obtained from the Genetic Modeling of Disease Centre (University of Manitoba), randomly sorted, and housed with maximum 5 mice per cage, in the central animal care facility at the University of Manitoba. Following acclimatization of one week, mice were challenged with intranasal (i.n.) administrations of 25 µg (35 µL of 0.7 mg/mL saline) of HDM protein extract (Greer Laboratories, Lenoir, NC, USA), daily for five consecutive days per week for two weeks. HDM used in this study was with low endotoxin content (< 300 EU/mg protein weight). HDM instillations were performed in the morning between 9 am and noon. Mice were visually monitored daily for grooming and activity. BALF was collected 24 h after the last HDM challenge based on our previous studies [26, 33]. Mice were anesthetized using sodium pentobarbital followed by tracheostomy in which a cannula was inserted into the trachea and lung was washed twice, each time with 1 mL (total 2 mL) of cold saline to obtain BALF samples.

### Cytokine profiling in bronchoalveolar lavage fluid and lung tissue

 BALF samples were centrifuged (150xg for 10 min) to obtain cell-free supernatant. BALF (50 µL) was used for cytokine assessment. Lung tissue specimen from the right lung middle lobe was collected in Tissue Protein Extraction Reagent T-Per (Pierce; Thermofisher Scientific, Rockford, IL, USA) containing Protease Inhibitor Cocktail (Sigma Aldrich, Oakville, ON, Canada). Tissue was homogenized on ice using the Cole-Parmer LabGEN 125 Homogenizer (Canada Inc, Montreal, QC, Canada). Homogenates were centrifuged (10,000 x g) to obtain tissue lysate. Protein amount in the tissue lysate was quantified with bicinchoninic acid (BCA) Protein Assay (Pierce). Total protein (50 µg) was used from each lung tissue lysate for cytokine evaluation. BALF and lung tissue lysates were aliquoted and stored at -20 °C until used. Abundance of a panel of murine cytokines and chemokines was measured in BALF and lung tissue lysates using the V-plex Mouse Cytokine 29-Plex Kit and the multiplex Meso Scale Discovery (MSD) platform (Meso Scale Discovery, Rockville, MD, USA), as per the manufacturer’s instructions. Data was analyzed using the Discovery Workbench 4.0 software (Meso Scale Discovery).

## Supplementary Information

**Additional file 1**

## Data Availability

All datasets used and/or analysed during the study are available from the corresponding author on reasonable request.

## Abbreviations

ALI: Air-Liquid Interface
AHR: Airway hyperresponsiveness
BALF: Bronchoalveolar lavage fluid
MAPK: ERK mitogen-activated protein kinase
HDM: House dust mite
HDP: Host defence peptides
HBEC: Human bronchial epithelial cells
IPA: Ingenuity Pathway Analysis
IL: Interleukin
LCN-2: Lipocalin 2
MEK: MAPK/ERK kinase
MMP13: Matrix metalloproteinase 13
MSD: Meso Scale Discovery
NET: Neutrophil-Extracellular Trap
PI3K: Phosphoinositide 3-kinase
PBEC: Primary bronchial epithelial cells
PKC: Protein kinase-C
Th: T-helper
TC: Tissue culture
