# Supplementary material for: Combination of IL-17A/F and TNF-α uniquely alters the bronchial epithelial cell proteome to enhance proteins that augment neutrophil migration
Source: J Inflamm (Lond). 2022 Dec 14;19:26. doi: 10.1186/s12950-022-00323-w (PMC9749191; doi:10.1186/s12950-022-00323-w)
Supplement: Supplementary file 1 — Additional file 1 [file 12950_2022_323_MOESM1_ESM.docx]

**SUPPLEMENTARY INFORMATION**

**FIGURES**

**
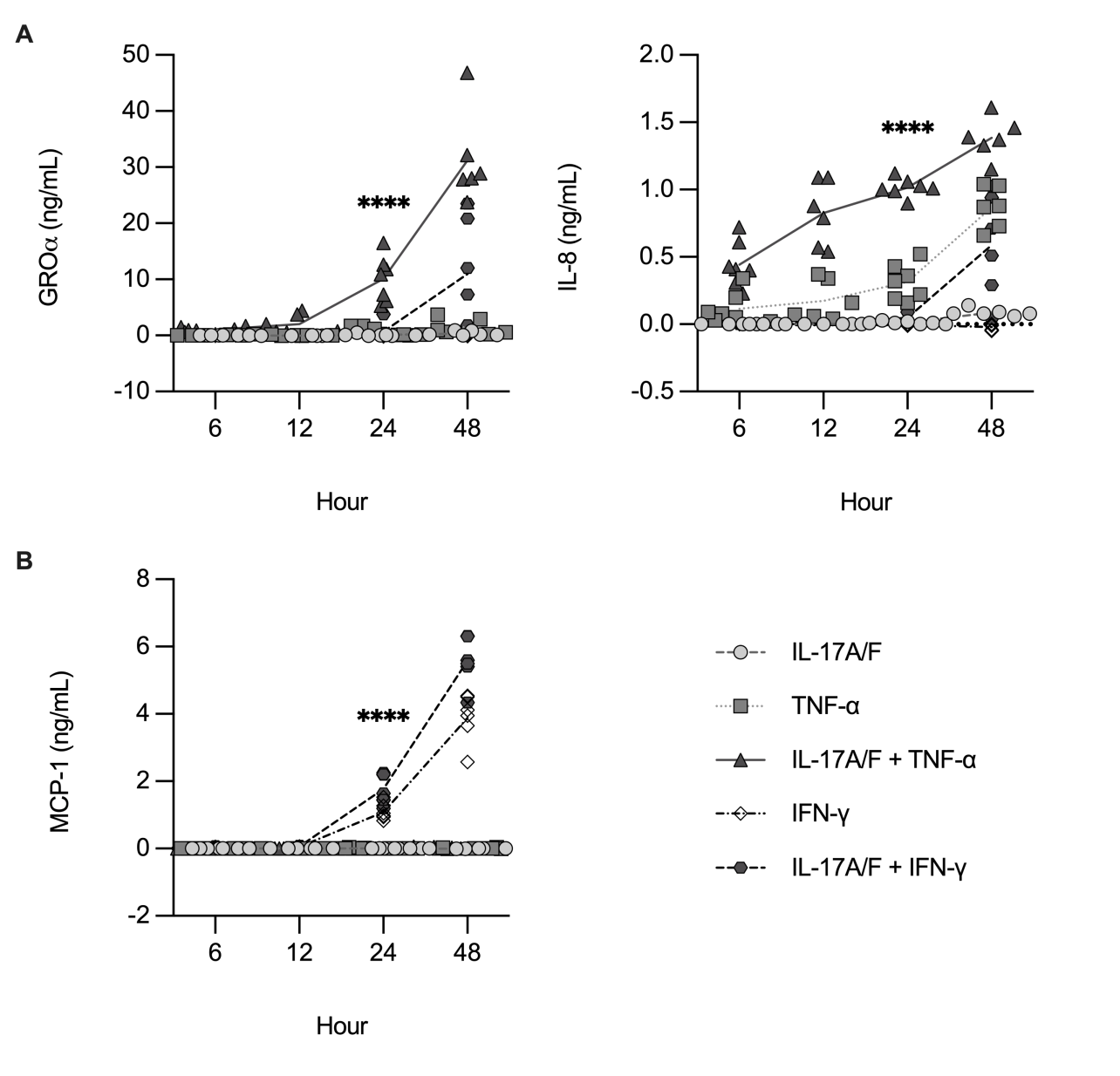
**

**Supplemental Figure 1: *Kinetic profile of protein production in human bronchial epithelial cells.*** HBEC-3KT cells were stimulated with either IL-17A/F (50 ng/mL), TNF-α (20 ng/mL), IFN-γ (30 ng/mL), or cytokine combinations as indicated, for 6, 12, 24, and 48 h. TC supernatants were examined by ELISA for secreted protein abundance of **(A)** IL-17A/F and/or TNF-α-associated neutrophil chemokines GROα and IL-8, and **(B)** IFN-γ-associated monocyte chemokine MCP-1. Increases in protein abundance are shown after subtraction of background values in paired unstimulated cell samples, for each replicate. Each data point represents an independent experimental replicate (N≥6), and lines represent the average. Fisher’s LSD test for two-way ANOVA was used to determine statistical significance of IL-17A/F-mediated enhancement of acute pro-inflammatory cytokines TNF-α and IFN-γ (*****p<0.0001*).

**
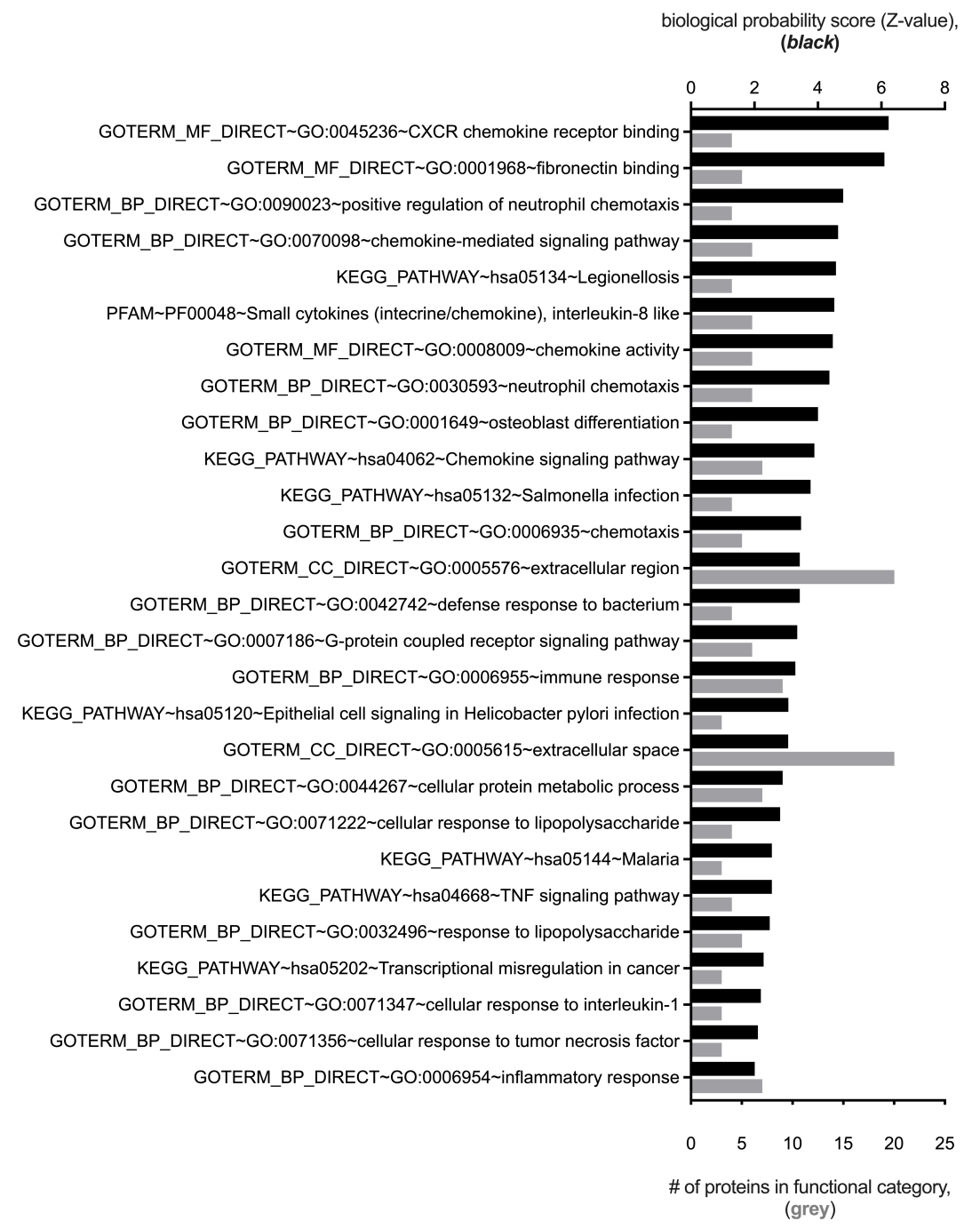
**

**Supplemental Figure 2: *Pathway enrichment analysis.*** HBEC-3KT cells were stimulated with IL-17A/F (50 ng/mL) in the presence/absence of TNF-α (20 ng/mL) for 24 hours. Cell lysates (14 µg total protein per sample) obtained from five experimental replicates were probed independently using the high-content aptamer-based proteomic array. Pairwise differential analysis was conducted on normalized log2 protein expression values, and Welch’s t-test with a cut-off of *p<0.05* was used to select proteins that were significantly enhanced in response to the combination of IL-17A/F + TNF-α, compared to either cytokine alone. Statistically significant pathway enrichment was also determined by selecting positively up-regulated (>0.2 log2 protein abundance) proteins (n=30) using an in-house analytical tool developed to compute enrichment specific to the SOMAmer®-specific collection of >1300 proteins. The enrichment score represents the probability that the submitted collection of proteins would occur within a given biological process due to randomness.

**
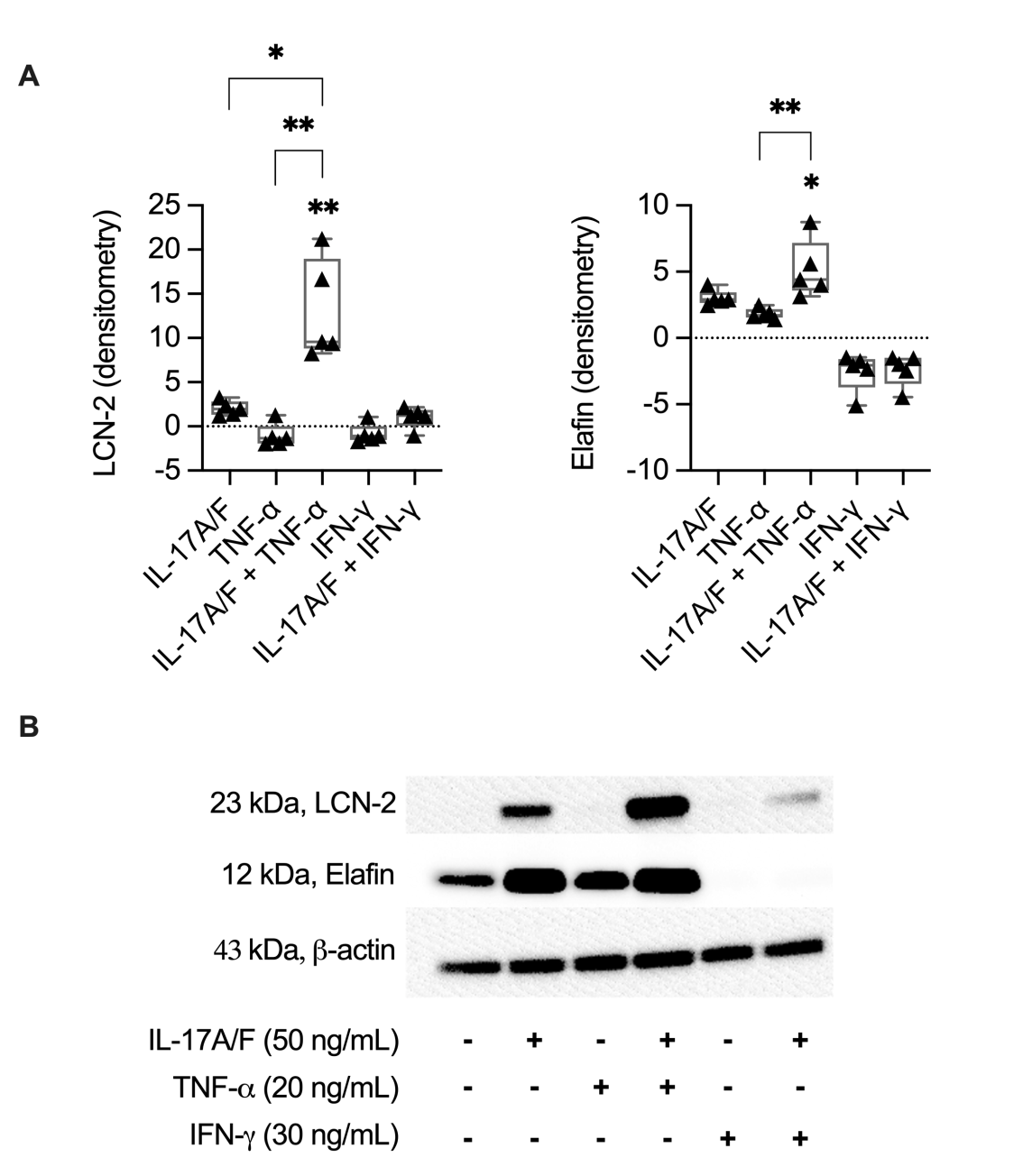
**

**Supplementary Figure 3: *Lipocalin-2 and Elafin protein production is synergistically enhanced in human bronchial epithelial cell lysate.*** HBEC-3KT were stimulated with either IL-17A/F (50 ng/mL), TNF-α (20 ng/mL), IFN-γ (30 ng/mL), or cytomix as indicated, for 24 h. Equivalent loading of cytosolic cell lysates (10 µg per sample) were monitored for the protein abundance of LCN-2 and Elafin by Western blot. Changes in protein abundance are shown as the ratio of abundance in cytokine-treated cells compared to unstimulated cells, for each replicate. The dashed lines represent baseline value from unstimulated cells. **(A)** Results are shown as boxplots, wherein bars show median and IQR, and whiskers show minimum and maximum values. Each data point represents an independent experimental replicate (N=5). **(B)** Representative blot. Fisher’s LSD test for one-way ANOVA was used to determine statistical significance (**p<0.05, **p<0.01*).


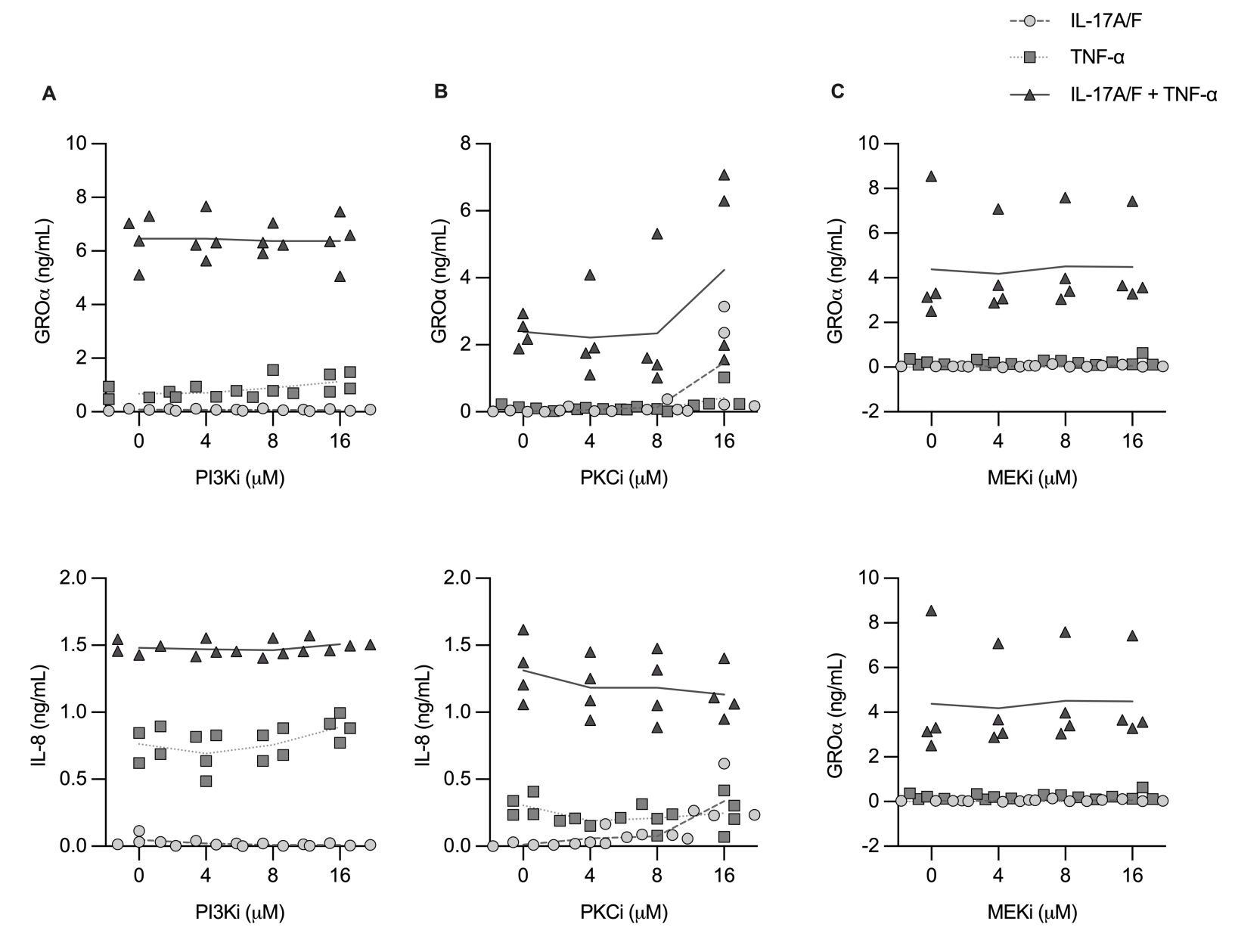


**Supplementary Figure 4: *Assessment of pharmacological inhibitors on IL-17A/F and TNF-α mediated production of selected neutrophil chemokines.*** HBEC-3KT cells were pre-treated with pharmacological inhibitors **(A)** LY294002 (PI3Ki), **(B)** GO6976 (PKCi), and **(C)** PD98059 (MEKi), for 1 h prior to stimulation with IL-17A/F (50 ng/mL), TNF-α (20 ng/mL) or the combination of IL-17A/F and TNF-α. TC supernatants were collected after 24 h and examined for GROα and IL-8 abundance by ELISA. Protein abundance shown is after subtraction of background abundance in paired unstimulated cells from each independent replicate. Each data point represents results from an independent experimental replicate (N=4) and the line represents the average. Increase in neutrophil migration was calculated after subtraction of neutrophil numbers found with tissue culture supernatant from paired unstimulated cells in trans-well migration assay, for each biological replicate. Two-way ANOVA with Dunnett’s test for multiple comparisons was used to determine statistical significance.


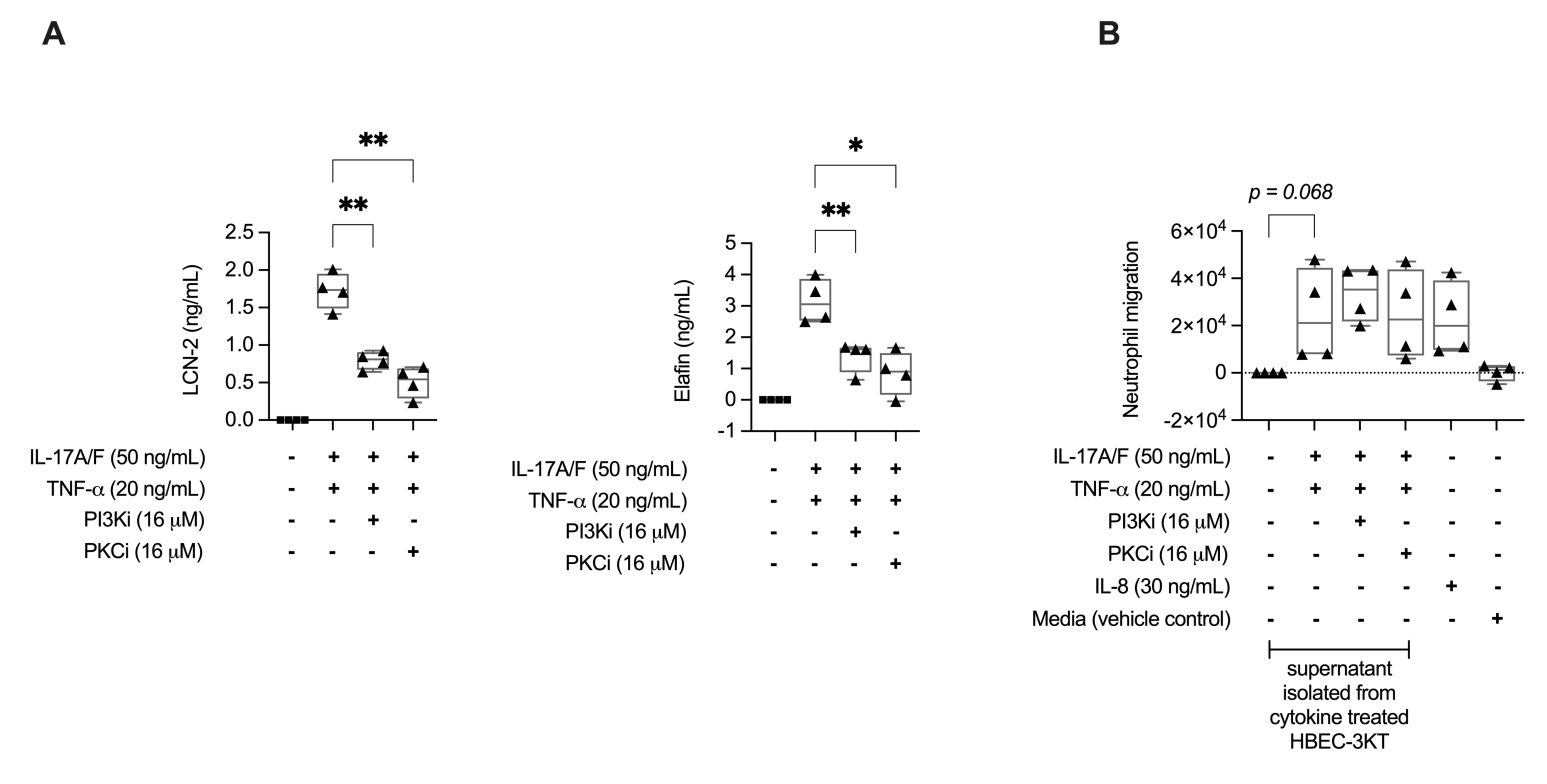


**Supplementary Figure 5: *Functional validation of neutrophil migration enhanced by the combination of IL-17A/F and TNF-α, in the presence/absence of selected pharmacological inhibitors.*** HBEC-3KT cells were stimulated with combination of IL-17A/F (50 ng/mL) and TNFα (20 ng/mL) in the presence and absence of pharmacological inhibitors LY294002 (PI3Ki; 16 uM) and GO6976 (PKCi; 16 uM) as indicated. Tissue culture supernatants collected after 24 h stimulation were **(A)** examined for the abundance of LCN-2 and Elafin by ELISA, and **(B)** used in bottom chamber of trans-well plates to examine the migration of neutrophils isolated from human blood. **(A)** Each data point represents results of an independent experimental replicate (N=4). Results are shown as boxplots, wherein bars show median and IQR, and whiskers show minimum and maximum value. Fisher’s LSD test for one-way analysis of variance (ANOVA) was used to determine statistical significance. **(B)** Cell culture medium spiked with human recombinant IL-8 (30 ng/ml) was used as a positive control, and medium alone was used as a vehicle control. Results are shown as boxplots, wherein bars show median and IQR, and whiskers show minimum and maximum value. Each data point represents the average number of neutrophils that traversed the membrane within two hours in each independent experimental replicate (N=4). One-way ANOVA with Bonferroni’s post-hoc test for multiple comparisons was used to determine statistical significance (**p*<0.05, ***p*<0.01).

**Supplementary Table 1: Proteins significantly altered in response to the combination of IL-17A/F and TNF-α, compared to either cytokine alone.**

| **Protein** | | **Average Log2 Fold Change**  **(Normalized to unstimulated cell)** | | | **Average Log2 Fold Change**  **(IL-17A/F + TNFα vs.**  **IL-17A/F or TNFα)** | |
| --- | --- | --- | --- | --- | --- | --- |
| **NAME** | **SWPROT ID** | **IL-17A/F** | **TNFα** | **IL-17A/F + TNFα** | **DIFFERENCE** | **P-SCORE** |
| **LCN-2** | P80188 | 1.86 | -0.31 | 4.05 | 3.28 | 8.06E-06 |
| **IL-8** | P10145 | 0.07 | 0.53 | 3.25 | 2.95 | 3.09E-06 |
| CFB | P00751 | 0.15 | 1.69 | 3.37 | 2.45 | 7.19E-06 |
| **GROα** | P09341 | 0.12 | 0.26 | 1.73 | 1.54 | 2.13E-05 |
| STC1 | P52823 | 1.27 | 0.90 | 2.59 | 1.50 | 3.61E-08 |
| **ELAFIN** | P19957 | 1.64 | 1.14 | 2.63 | 1.24 | 5.73E-04 |
| **MMP13** | P45452 | -0.19 | 1.51 | 1.82 | 1.17 | 3.04E-03 |
| IGFBP5 | P24593 | 0.74 | -0.11 | 1.30 | 0.98 | 1.29E-03 |
| IGFBP3 | P17936 | 0.57 | -0.01 | 1.09 | 0.81 | 9.29E-05 |
| TNC | P24821 | 0.14 | 1.35 | 1.39 | 0.65 | 1.70E-02 |
| PPBP | P02775 | -0.06 | -0.12 | 0.48 | 0.57 | 1.76E-04 |
| ARID3A | Q99856 | 0.03 | 0.25 | 0.65 | 0.51 | 2.22E-04 |
| PLAUR | Q03405 | 0.01 | 1.13 | 1.02 | 0.46 | 4.96E-02 |
| SLPI | P03973 | 0.41 | 0.43 | 0.88 | 0.46 | 3.34E-04 |
| CD55 | P08174 | 0.09 | 0.32 | 0.63 | 0.42 | 1.46E-03 |
| LYN | P07948 | -0.03 | 0.64 | 0.72 | 0.41 | 3.40E-02 |
| CCL20 | P78556 | 0.16 | 0.34 | 0.63 | 0.38 | 3.67E-03 |
| S100A9 | P06702 | 0.10 | -0.01 | 0.39 | 0.34 | 2.19E-04 |
| FSTL1 | Q12841 | -0.02 | 0.30 | 0.45 | 0.31 | 4.55E-03 |
| HK2 | P52789 | 0.19 | 0.53 | 0.67 | 0.31 | 2.50E-02 |
| CSF3 | P09919 | 0.04 | 0.05 | 0.36 | 0.31 | 4.48E-04 |
| CXCL3 | P19876 | 0.14 | 0.19 | 0.47 | 0.30 | 9.66E-04 |
| CXCL2 | P19875 | 0.14 | 0.19 | 0.47 | 0.30 | 9.66E-04 |
| CTSS | P25774 | 0.04 | 0.61 | 0.62 | 0.29 | 2.24E-02 |
| CDH1 | P12830 | 0.07 | -0.16 | 0.21 | 0.26 | 2.60E-02 |
| MICA | Q29983 | -0.05 | 0.26 | 0.34 | 0.24 | 4.89E-03 |
| SAA1 | P0DJI8 | 0.17 | 0.18 | 0.41 | 0.23 | 1.01E-03 |
| ROBO2 | Q9HCK4 | 0.01 | 0.45 | 0.46 | 0.23 | 2.33E-02 |
| MET | P08581 | -0.02 | -0.11 | 0.15 | 0.21 | 4.87E-05 |
| FSTL3 | O95633 | 0.01 | 0.19 | 0.31 | 0.21 | 3.43E-02 |
| HBEGF | Q99075 | -0.07 | 0.06 | 0.19 | 0.19 | 2.49E-02 |
| MICB | Q29980 | 0.04 | 0.23 | 0.30 | 0.17 | 3.32E-02 |
| CXCL16 | Q9H2A7 | 0.09 | 0.19 | 0.30 | 0.15 | 1.42E-02 |
| NTN4 | Q9HB63 | 0.09 | -0.06 | 0.17 | 0.15 | 2.19E-02 |
| FGF2 | P09038 | -0.03 | -0.03 | 0.10 | 0.13 | 2.68E-02 |
| FGFR2 | P21802 | 0.00 | 0.01 | 0.07 | 0.06 | 2.32E-02 |
| SIRPA | P78324 | -0.03 | 0.01 | 0.03 | 0.05 | 2.27E-02 |
| AGRP | O00253 | 0.02 | 0.03 | 0.05 | 0.02 | 4.59E-02 |
| PDGFRA | P16234 | 0.06 | 0.04 | 0.01 | -0.04 | 1.47E-02 |
| C8A | P07357 | 0.01 | 0.02 | -0.03 | -0.05 | 3.67E-02 |
| C8B | P07358 | 0.01 | 0.02 | -0.03 | -0.05 | 3.67E-02 |
| C8G | P07360 | 0.01 | 0.02 | -0.03 | -0.05 | 3.67E-02 |
| COL18A1 | P39060 | -0.07 | -0.13 | -0.16 | -0.06 | 2.06E-02 |
| LTBR | P36941 | -0.01 | -0.03 | -0.08 | -0.06 | 4.46E-02 |
| CTSZ | Q9UBR2 | -0.06 | -0.02 | -0.10 | -0.06 | 1.04E-02 |
| C2 | P06681 | -0.06 | -0.07 | -0.14 | -0.07 | 1.97E-03 |
| PSMA6 | P60900 | -0.02 | -0.02 | -0.10 | -0.08 | 1.04E-02 |
| IGFBP6 | P24592 | -0.08 | -0.14 | -0.20 | -0.09 | 2.32E-02 |
| IL6 | P05231 | -0.14 | -0.16 | -0.25 | -0.10 | 1.24E-02 |
| C10orf54 | Q9H7M9 | 0.00 | -0.18 | -0.20 | -0.11 | 3.79E-02 |
| COL23A1 | Q86Y22 | -0.13 | -0.18 | -0.28 | -0.12 | 1.90E-03 |
| CDC2 | P06493 | 0.00 | -0.12 | -0.19 | -0.13 | 2.05E-02 |
| MPO | P05164 | -0.14 | -0.19 | -0.30 | -0.14 | 1.75E-03 |
| LTBP4 | Q8N2S1 | -0.12 | -0.38 | -0.39 | -0.15 | 3.76E-02 |
| LRP8 | Q14114 | -0.13 | -0.18 | -0.32 | -0.17 | 3.50E-04 |
| SNX4 | O95219 | -0.09 | -0.10 | -0.28 | -0.18 | 3.24E-02 |
| SPARC | P09486 | -0.08 | -0.03 | -0.25 | -0.20 | 6.71E-04 |
| EREG | O14944 | -0.16 | 0.11 | -0.24 | -0.22 | 2.87E-02 |
| LAMA1 | P25391 | -0.21 | -0.52 | -0.64 | -0.27 | 4.00E-03 |
| LAMB1 | P07942 | -0.21 | -0.52 | -0.64 | -0.27 | 4.00E-03 |
| LAMC1 | P11047 | -0.21 | -0.52 | -0.64 | -0.27 | 4.00E-03 |
| ECM1 | Q16610 | -0.25 | -0.65 | -0.73 | -0.28 | 2.39E-02 |
| THBS1 | P07996 | -0.15 | 0.13 | -0.35 | -0.34 | 7.22E-03 |
| NRP1 | O14786 | -0.30 | -1.11 | -1.11 | -0.40 | 2.73E-02 |
| EFEMP1 | Q12805 | -0.17 | -0.39 | -0.70 | -0.42 | 1.02E-02 |
| FTH1 | P02794 | -0.27 | -1.40 | -1.40 | -0.56 | 2.43E-02 |
| FTL | P02792 | -0.27 | -1.40 | -1.40 | -0.56 | 2.43E-02 |
| LRIG3 | Q6UXM1 | -0.36 | -1.20 | -1.37 | -0.59 | 6.05E-03 |
| MMP1 | P03956 | -0.42 | 0.57 | -0.69 | -0.77 | 9.81E-03 |
| CTSV | O60911 | -0.57 | -1.88 | -2.15 | -0.93 | 2.86E-03 |
